# Hub gene identification and molecular subtype construction for *Helicobacter pylori* in gastric cancer via machine learning methods and NMF algorithm

**DOI:** 10.18632/aging.205053

**Published:** 2023-09-26

**Authors:** Lianghua Luo, Ahao Wu, Xufeng Shu, Li Liu, Zongfeng Feng, Qingwen Zeng, Zhonghao Wang, Tengcheng Hu, Yi Cao, Yi Tu, Zhengrong Li

**Affiliations:** 1 Department of General Surgery, The First Affiliated Hospital of Nanchang University, Nanchang, Jiangxi, China; 2 Medical Innovation Center, The First Affiliated Hospital of Nanchang University, Nanchang, Jiangxi, China; 3 Department of Pathology, The First Affiliated Hospital of Nanchang University, Nanchang, Jiangxi, China

**Keywords:** gastric cancer, *Helicobacter pylori*, cluster, hub genes, therapy

## Abstract

*Helicobacter pylori* (HP) is a gram-negative and spiral-shaped bacterium colonizing the human stomach and has been recognized as the risk factor of gastritis, peptic ulcer disease, and gastric cancer (GC). Moreover, it was recently identified as a class I carcinogen, which affects the occurrence and progression of GC via inducing various oncogenic pathways. Therefore, identifying the HP-related key genes is crucial for understanding the oncogenic mechanisms and improving the outcomes of GC patients. We retrieved the list of HP-related gene sets from the Molecular Signatures Database. Based on the HP-related genes, unsupervised non-negative matrix factorization (NMF) clustering method was conducted to stratify TCGA-STAD, GSE15459, GSE84433 samples into two clusters with distinct clinical outcomes and immune infiltration characterization. Subsequently, two machine learning (ML) strategies, including support vector machine-recursive feature elimination (SVM-RFE) and random forest (RF), were employed to determine twelve hub HP-related genes. Beyond that, receiver operating characteristic and Kaplan-Meier curves further confirmed the diagnostic value and prognostic significance of hub genes. Finally, expression of HP-related hub genes was tested by qRT-PCR array and immunohistochemical images. Additionally, functional pathway enrichment analysis indicated that these hub genes were implicated in the genesis and progression of GC by activating or inhibiting the classical cancer-associated pathways, such as epithelial-mesenchymal transition, cell cycle, apoptosis, RAS/MAPK, etc. In the present study, we constructed a novel HP-related tumor classification in different datasets, and screened out twelve hub genes via performing the ML algorithms, which may contribute to the molecular diagnosis and personalized therapy of GC.

## INTRODUCTION

Gastric cancer (GC) is one of the most frequent and fatal upper gastrointestinal malignances, all ranking the top five among cancers globally in terms of the morbidity and mortality [1]. According to the World Health Organization (WHO), the incidence and mortality rate of GC have gradually declined, partly due to increased awareness of *Helicobacter pylori* (HP) infection [2]. Nevertheless, when patients with GC are diagnosed at a late phase or metastasis, they are usually linked with dismal clinical outcomes, with < 30% 5-year survival rate [3]. Consequently, it is imperative to identify more valuable and accurate novel biomarkers to improve the early diagnosis and therapeutic avenues of GC.

Over half of the world’s population is infected by HP, a spiral-shaped, gram-negative, microaerophilic bacterium that preferentially colonizes the human gastric mucosa [4]. Due to the majority of HP-positive individuals being asymptomatic or having subtle symptoms, it was able to elude researchers’ notice. It has progressively come to light that HP infection may predispose a person to a variety of stomach diseases, including atrophic gastritis, peptic ulcers, and even GC, until Barry Marshall and Robin Warren debunk it [5–8]. Recent research has shown a direct link between HP infection and GC, with individuals with HP positivity experiencing three to six times as many cases as those with HP negativity [9, 10]. Beyond that, HP infection may limit the death of tumor cells and promote gastric carcinogenesis in addition to methylating many cancer-associated genes on CpG islands in gastric epithelial cells [11–14]. The International Agency for Research on Cancer (IARC) recently classified HP as a classification I biological carcinogen, and it has the potential to cause GC by one of the following three mechanisms: DNA damage to epithelial cells, a reduction in repair activity, a mitochondrial DNA mutation, and the emergence of transitory mutation phenotypes are all examples of this [15, 16].

Machine learning (ML), one of the most significant subfields of artificial intelligence (AI), has been extensively utilized in a variety of biomedical domains, including disease diagnosis, biomarker identification, and drug discovery, among others [17, 18]. Moreover, a number of well-known ML techniques, such random forest (RF) and support vector machine (SVM) with recursive feature elimination (RFE), have made significant progress in the creation of anti-cancer drugs and the diagnosis of complicated diseases [19–21].

In this study, based on the differentially expressed HP-related genes, non-negative matrix factorization (NMF) clustering approach was applied to sort The Cancer Genome Atlas (TCGA) stomach adenocarcinoma (STAD), GSE15459, GSE84433 cohorts into two molecular subtypes with different prognosis, immune infiltration landscape, and anticancer drug sensitivity, indicating that the HP-related genes were closely related to the clinical outcomes and therapeutic efficacy of GC. Then, two classical ML algorithms, were employed to identify twelve HP-related hub genes, namely, EFNA3, UHRF1, FLT1, NRP1, CTLA4, L3MBTL3, MAPK10, MLEC, MYL9, THY1, MYB, and NCLN. Subsequently, receiver operating characteristic (ROC) and Kaplan-Meier (K-M) curves were utilized to examine the diagnostic and prognostic performance of these hub genes, and we also explored the anticancer drug sensitivity, immune cell infiltration and mutational features of the hub genes. Finally, quantitative reverse transcription polymerase chain reaction (qRT-PCR) experiments and immunohistochemical (IHC) images from online browsers were exploited to verify the differential expression of these twelve hub genes.

## MATERIALS AND METHODS

### Data acquisition and pre-processing

The transcriptome profiles and corresponding clinical information of GC patients in the present study were retrieved and downloaded from the TCGA portal (https://portal.gdc.cancer.gov/, up to May 18, 2022) and GEO database (https://www.ncbi.nlm.nih.gov/geo/, up to May 18, 2022). After filtering out some cases without survival time and status, the ComBat method was employed to correct the batch effects of raw sequencing data sets from different platforms for ensuring comparability among all samples. In the meantime, the somatic mutation and copy number variation (CNV) data of GC patients in TCGA were obtained via querying the UCSC Xena browser (http://xena.ucsc.edu/, up to May 18, 2022), and the “MAFtools” and “RCircos” packages in R software (version 4.1.0, https://www.r-project.org/) were applied to summarize and visualize these data. In addition, the list of HP-related gene sets was collected from the Molecular Signatures Database (MSigDB) (http://www.broadinstitute.org/gsea/msigdb).

### NMF consensus clustering analysis

Non-negative matrix factorization (NMF) algorithm is a non-negative factorization of a matrix under the condition that all the elements of the matrix are non-negative, so as to find out the relationships and interactions between them. Elements with similar characteristics are grouped into one group and elements with different characteristics are grouped into another group. Prior to performing the NMF clustering algorithm, the “limma” package with the thresholds of p < 0.05 and |logFC| (fold change) > 1 was utilized to screen out the differentially expressed HP-related genes. Afterwards, on the basis of these genes, the NMF clustering method was implemented to sort TCGA, GSE15459, GSE84433 samples into different clusters by using the “NMF” package, respectively. The number of clusters (K) from 2 to 10 were tested by running ten iterations per K, and the optimal K number was eventually determined in accordance with silhouette, consensus, as well as cophenetic. Principal component analysis (PCA) was used to detect the classification capability of clusters. Then, Kaplan-Meier (K-M) curve was carried out to estimate the differences of OS between distinct subtypes. Moreover, the CIBERSORT and ESTIMATE algorithms were performed to elucidate the immune infiltration landscape of different clusters. According to the half-maximal inhibitory concentration (IC50) value, the “pRRophetic” package was conducted to explore the sensitivity and resistance of common anticancer agents (e.g., Imatinib, Cisplatin) across different clusters. Finally, we also evaluated the expression level of five common immune checkpoint blockade-associated genes (i.e., PDCD1, BTLA, CTLA4, CD274, PD-L2) across different GC subtypes.

### Identification of the HP-related hub genes via ML strategies

As it is well known, HP infection is clearly associated with the majority of GC patients [22]. To find out more deeply the impact of HP-related genes on GC patient prognosis, univariate Cox regression analysis (p < 0.05) was exploited to screen the prognostic-associated HP genes prior to implementing ML strategies. ML methods displayed excellent and robust performance compared to traditional means, especially in disease diagnosis and predictive analytics [23, 24]. As the two most prevalent supervised ML algorithms, RF and SVM-RFE, were carried out in this study to identify the HP-related hub genes. The establishment of the SVM-RFE model mainly depends on the “e1071”, “kernlab”, and “caret” packages, and the RF classifier is performed via running the “randomForest” package.

### Assessment of diagnostic performance and prognostic value

The area under the ROC curve (AUC) is the gold standard metric in diagnostic performance evaluation, which is calculated through executing the “pROC” package [25]. To obtain into a more convenient clinical application of the HP-related hub genes, a nomogram based on the TCGA-STAD cohort was built to assist with the diagnosis of GC via using the “rms” package. Furthermore, decision, clinical impact, as well as calibration curves were created to examine the sensitivity and accuracy of the diagnosis nomogram.

To investigate the prognostic value of the HP-related hub genes, we categorized all TCGA-STAD patients into high- or low- expression subgroups in accordance with the median expression values of these genes, separately. Next, the K-M method and log-rank test were adopted to confirm the effects of these hub genes on GC prognosis. Apart from this, we also used another survival analysis approach (univariate COX analysis) to discern whether these hub genes were protective or risk factors in the clinical outcomes of GC patients. K-M Plotter is an online website (http://kmplot.com/analysis/) containing a large amount of GEO GC datasets (GSE14210(N=145), GSE15459(N=200), GSE22377(N=43), GSE29272(N=268), GSE51105(N=94), GSE62254(N=300)), which is able to rapidly estimate the prognostic effects of genes. To demonstrate that the prognostic value of hub genes was not just confined to the TCGA cohort, KM Plotter was applied to further validate their broad applicability in numerous datasets from other sources.

### Characteristics of infiltrating immune cells

To reveal the immune-infiltrating landscape of TCGA-STAD samples, the CIBERSORT program was utilized to estimate the relative abundances of 22 types of infiltrating immune cells in each sample by using the “CIBERSORT” package [26, 27]. Furthermore, Spearman’s correlation analysis was performed to demonstrate the correlation between the expression levels of these hub genes and tumor-infiltrating immune cells.

### Drug response prediction

By logging in to the Gene Set Cancer Analysis (GSCA) (http://bioinfo.life.hust.edu.cn/GSCA/#/drug) database, the association between the sensitivity of drugs derived from the Genomics of Drug Sensitivity in Cancer (GDSC) database and the mRNA expression of hub genes was explored [28]. At the same time, the chemical structural formulas of GDSC agents that exhibited a significant positive or negative correlation with the expression of these hub genes were collected by querying the MedChemExpress website (https://www.medchemexpress.cn/).

### Functional and pathway enrichment analysis

To assess the interaction among hub genes, a protein–protein interaction (PPI) network was created to examine how closely they were connected via using the GeneMANIA (http://genemania.org/) online tool. Moreover, an interaction map between hub genes and cancer-associated pathways was constructed to further explore the biological role of HP-related hub genes in the onset and progression of GC by making use of the GSCALite (http://bioinfo.life.hust.edu.cn/web/GSCALite/). Gene set enrichment analysis (GSEA), another powerful method for functional enrichment of gene sets, was carried out to probe the biological functions of hub genes. Investigating the upstream regulated miRNAs is essential to gain insight into the mechanisms of action of genes, and a miRNA-mRNA regulatory network for hub genes was also predicted and generated through the GSCALite website.

### Validation of hub gene expression

For mRNA expression levels of hub genes, qRT-PCR experiments were conducted in accordance with manufacturers’ instructions and our previous study [29]. A normal gastric epithelial cell line (GES-1) and five human GC cell lines (HGC-27, AGS, MKN-45, MGC803, and MKN-28) were obtained from the Shanghai Cell Bank of the Chinese Academy of Sciences. The glyceraldehyde-3-phosphate dehydrogenase (GAPDH) was treated as internal reference. The 2−ΔΔCq method was adopted to calculate the relative expression levels of hub genes, and GraphPad Prism 6.0 software was utilized to plot bar graphs. Apart from this, the mRNA primer sequences of hub genes were summarized in detail (Supplementary Table 1).

The Human Protein Atlas (HPA) (http://www.proteinatlas.org/) database provides large proteomics data of various normal tissues and corresponding cancers [30]. To verify the protein expression abundance of hub genes, immunohistochemical (IHC) staining images from HPA project were extracted for analysis and comparison.

### Data availability statement

The datasets presented in this study can be found in online repositories (TCGA, https://portal.gdc.cancer.gov/), (GEO, http://www.ncbi.nlm.nih.gov/geo/). The names of the repository/repositories and accession number(s) can be found in the article/Supplementary Material.

### Consent for publication

All contributors give consent for unrestricted publication of this work.

## RESULTS

### HP-related gene screening and functional enrichment analysis

**Figure 1:**
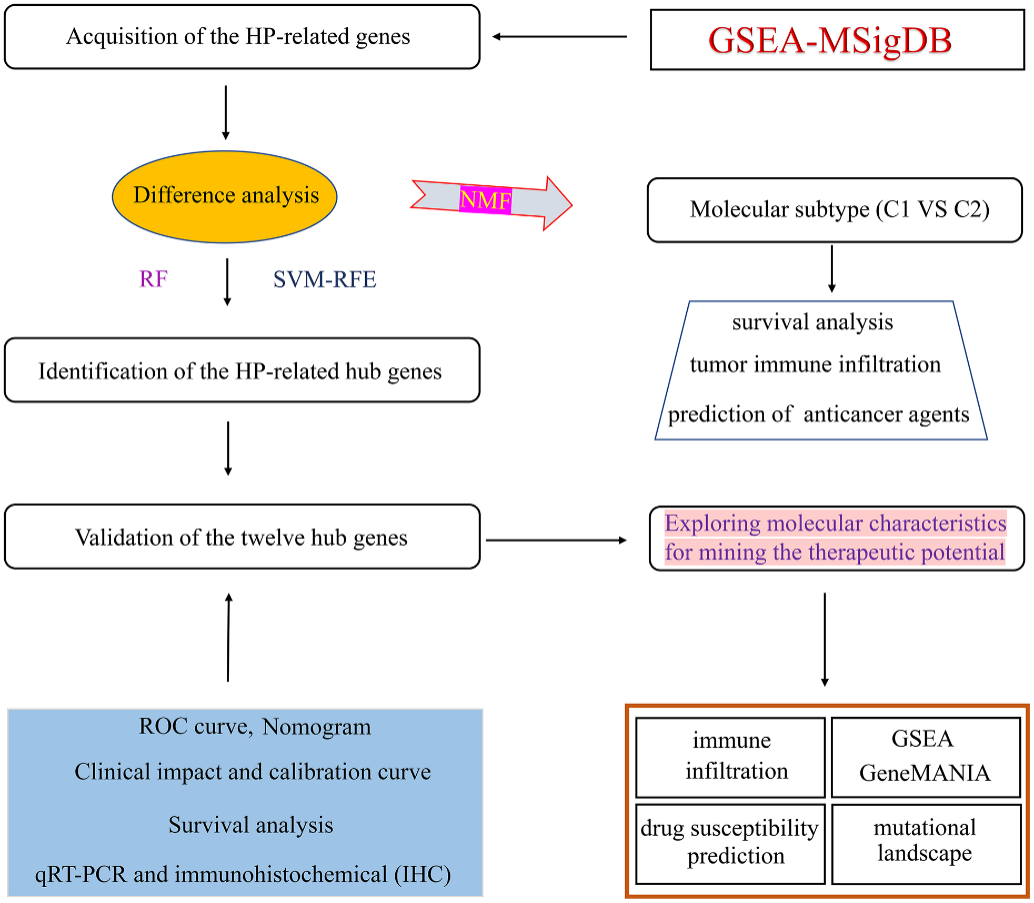
Flowchart illustrating the workflow of this study.

The flow-process diagram for this present research was summarized in Figure 1. Firstly, a total of 761 HP-related gene sets were extracted from the MSigDB website (Supplementary Table 2). Subsequently, differential expression analysis was carried out to screen 232 differentially expressed HP-related genes via using the “limma” package (Supplementary Table 3).

To further unravel the potential mechanisms of these genes in the occurrence and progression of GC, Gene Ontology (GO) functional and Kyoto Encyclopedia of Genes and Genomes (KEGG) pathway annotation analyses were executed by implementing the “clusterProfiler” package. GO analysis revealed that these genes were mainly enriched in cancer-associated biological functions, such as mitotic cell cycle, cell cycle, cell cycle process, chromosome organization, DNA replication, cell division, DNA conformation change, chromosomal region, condensed chromosome, heterochromatin, chromosome, ribonucleotide binding, DNA helicase activity, helicase activity, and integrin binding (Supplementary Figure 1A–1C). KEGG analysis disclosed the enriched top signaling pathways, including epithelial cell signaling in *Helicobacter pylori* infection, Vibrio cholerae infection, NOD-like receptor signaling pathway, pathways in cancer, spliceosome, Toll-like receptor signaling pathway, leukocyte transendothelial migration, as well as cytokine–cytokine receptor interaction (Supplementary Figure 1D).

### Establishment of molecular subtype based on the HP-related genes

NMF consensus clustering was conducted on the TCGA-STAD (N=338), GSE15459 (N=192), GSE84433 (N=357) cohorts respectively on the basis of 232 differentially expressed HP-related genes.

**Figure 2:**
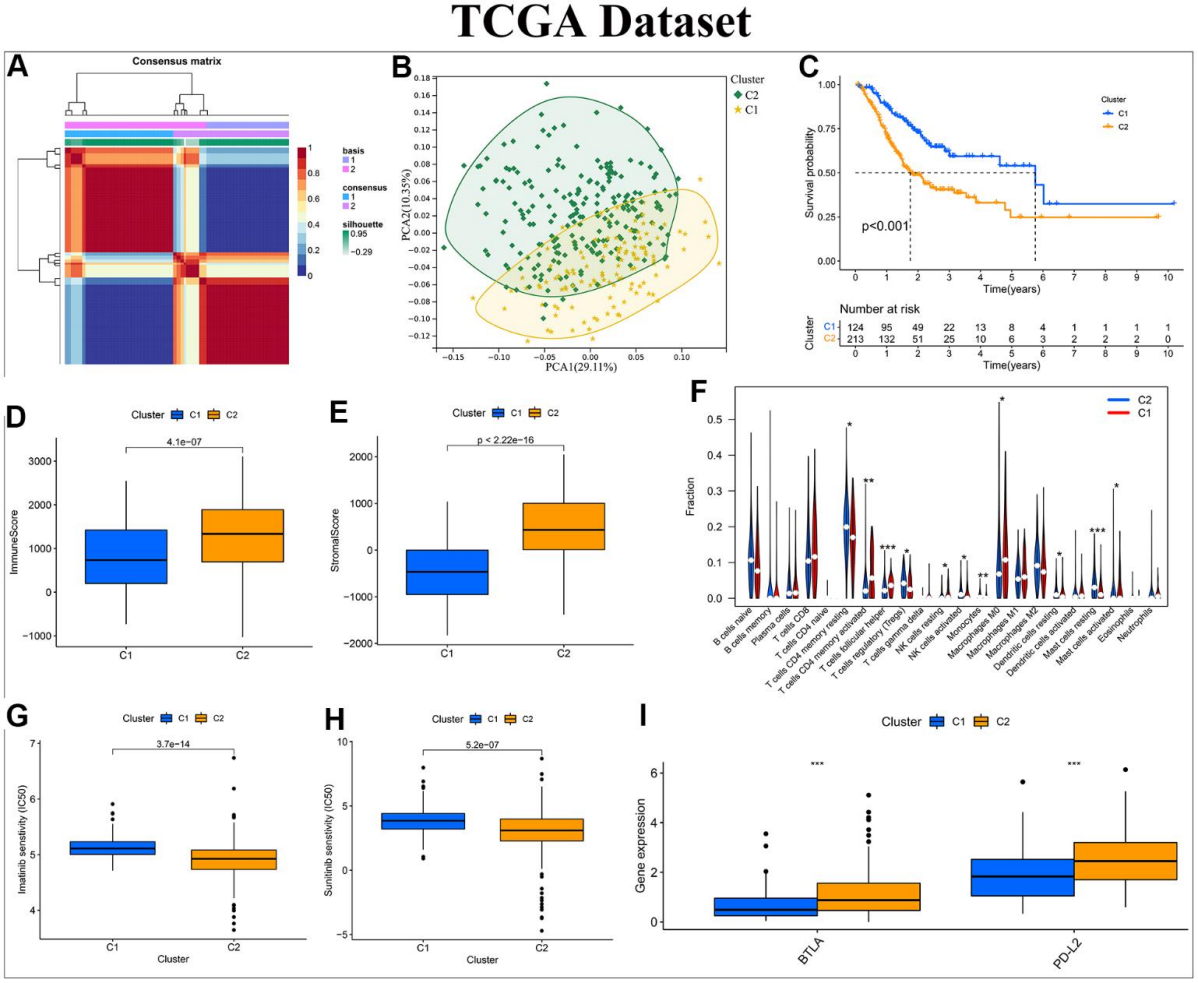
**Construction of a NMF subtype based on the differentially expressed HP-related genes in the TCGA-STAD cohort.** (**A**) NMF consensus clustering for k = 2. (**B**) Kaplan–Meier analysis of overall survival (OS) for Cluster C1 and C2. (**C**) Principal component analysis (PCA). (**D**, **E**) Differential analyses of immune and stromal score between Cluster C1 and C2. (**F**) Violin plot showing the immune cell infiltration landscape across different clusters. (**G**, **H**) Box plot of estimated IC50 values for Imatinib and Sunitinib in Cluster C1 and C2. (**I**) Box plot visualizing the significant expression differences of immune checkpoints across distinct clusters, including BTLA and PD-L2. *:P<0.05 ** :P<0.01 ***:P<0.001.

Following the prompts of silhouette, consensus, as well as cophenetic, all samples of the three different datasets were eventually split into 2 clusters (Figure 2A and Supplementary Figures 3A, 4A). The silhouette, consensus, and cophenetic heatmaps of each dataset were displayed in Supplementary Figure 2. PCA results suggested that clusters based on HP-related genes had excellent classification ability in distinct datasets (Figure 2B and Supplementary Figures 3B, 4B). Survival analysis also revealed that there was an obvious difference in the OS of patients between Cluster C1 and C2 in all datasets (Figure 2C and Supplementary Figures 3C, 4C). The tumor microenvironment (TME) has a close correlation with patient prognosis and tumor progression, and the immune and stromal cells constitutes the main components of TME [31]. The TME score files showed that patients with Cluster C1 yielded a lower immune score than that of C2, and the CIBERSORT algorithm was adopted to further explore the composition of immune cell infiltration between different clusters (Figure 2D, 2E and Supplementary Figures 3D, 3E, 4D, 4E). Notably, in the GSE84433 cohort, the infiltration abundance of immunosuppressive Tregs and Macrophages M2 in Cluster C1 was significantly higher than that in Cluster C2, which may be an important reason for the worse prognosis of patients with Cluster C1 (Supplementary Figure 3F). In contrast, in the TCGA-STAD cohort, patients in Cluster C1 exhibited a lower level of Tregs infiltration compared with that in Cluster C2, resulting in the better clinical outcomes for patients Cluster C1 (Figure 2F). Beyond this, in the GSE15459 cohort, a prevalent amplification in anti-tumorigenic immune cells, including NK cells resting, NK cells activated, Macrophages M0, Mast cells activated, Dendritic cells activated, was observed in Cluster C1, whereas a higher infiltration degree with Macrophages M2 was found in Cluster C2, which was a great explanation for the poorer survival rate of patients with Cluster C2 (Supplementary Figure 4F).

Considering that chemotherapy and targeted therapy are still served as a cornerstone of treatment for GC, we calculated the IC50 values of common anticancer agents in the two clusters to predict drug susceptibility or resistance. Immunotherapy has been an emerging therapeutic modality for GC, and the expression levels of five common immune checkpoint genes across different clusters were compared to predict the immunotherapeutic response of GC patients. Surprisingly, in the TCGA-STAD cohort, the IC50 values of imatinib and sunitinib were significantly lower in Cluster C2 than in Cluster C1, while the expression levels of BTLA and PD-L2 were obviously higher in C2 than in C1, all indicating that patients in the Cluster C2 were more likely to benefit from targeted therapy and immunotherapy to improve their dismal prognosis (Figure 2G–2I). Similarly, in the GSE84433 and GSE15459 cohorts, the expression levels of immune checkpoint genes and IC50 values of anticancer drugs across two clusters were also totally disparate (Supplementary Figures 3G–3I, 4G–4I).

Collectively, these results revealed that HP-related genes were closely related to the prognosis and treatment of GC patients, and the novel molecular subtype had great clinical practicality and wide applicability.

### ML identifying the HP-related hub genes

**Figure 3:**
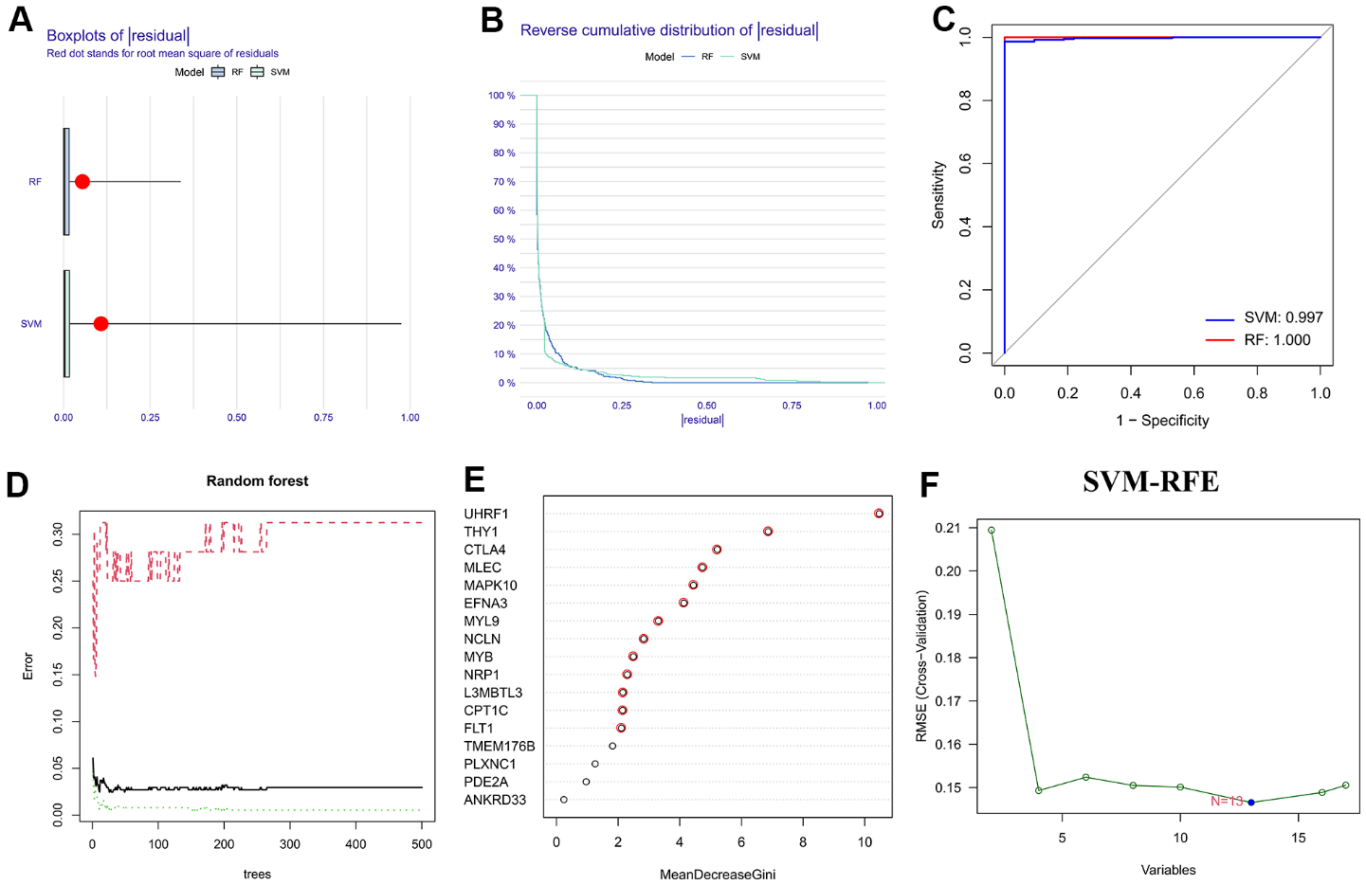
**Selection of the HP-related hub genes via machine learning strategies.** (**A**, **B**) Boxplot and reverse cumulative distribution curve of residual. (**C**) Comparison of ROC curves for evaluating the diagnostic reliability of support vector machine-recursive feature elimination (SVM-RFE) and random forest (RF) models. (**D**) Error graph of RF model. (**E**) Based on RF algorithm to screen the HP-related hub genes. (**F**) On the basis of SVM-RFE method to identify the HP-related hub genes.

**Figure 4:**
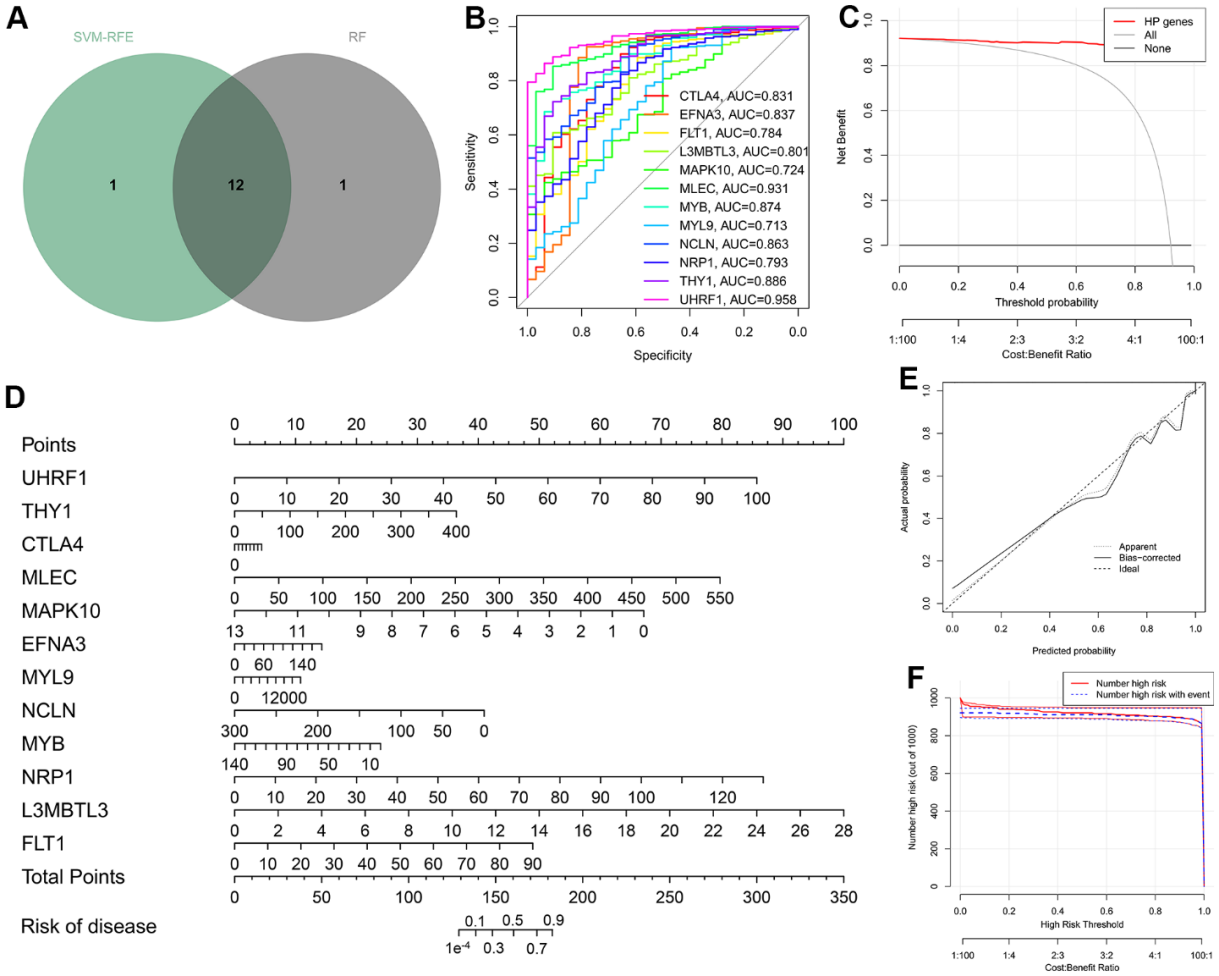
**Construction of the diagnostic nomogram on the basis of the twelve hub genes.** (**A**) Venn diagram taking the intersection of the results of two ML strategies. (**B**) ROC curves measuring the diagnostic efficacy of the twelve HP-related hub genes. (**C**) Decision curve of nomogram graph. (**D**) Nomogram for the diagnosis of gastric cancer (GC). (**E**) Calibration curve demonstrating the diagnostic performance of the nomogram. (**F**) Clinical impact curve.

Before implementing the ML algorithms, univariate Cox regression analysis was employed to obtain seventeen prognostic-associated HP genes from the above differentially expressed HP-related genes (Supplementary Table 4). Then, two classical ML methods, RF and SVM-RFE, were conducted to determine the HP-related hub genes. Based on the RNA-sequencing data of the seventeen prognostic-associated HP genes in TCGA-STAD dataset, thirteen candidate genes were identified through the feature selection of RF model (Figure 3D, 3E and Supplementary Table 5), and thirteen genes were acquired via implementing the SVM-RFE strategy (Figure 3F and Supplementary Table 6). Reverse cumulative distribution and boxplots plots showed that residual values of the two ML algorithms considered negligible (Figure 3A, 3B), and ROC curves demonstrated that both the SVM-RFE and RF models in this present study had a very robust accuracy score (SVM-RFE: 0.997, RF: 1) (Figure 3C). Therefore, by taking the intersection of the results of two ML strategies, twelve genes were determined and served as HP-related hub genes for follow-up analysis (Figure 4A).

### Evaluation of diagnostic performance and prognostic value of the twelve hub genes

To confirm the strong diagnostic power of the twelve hub genes, ROC curves were drawn by using the “pROC” package. All hub genes in the TCGA-STAD cohort reached the AUC values of 0.713–0.958, of which UHRF1 achieved the highest AUC value of 0.958 (Figure 4B). To better make use of these hub genes, a nomogram containing all hub genes was established for the diagnosis of GC through the “rms” package (Figure 4D). The well-calibrated capability of the nomogram was observed by checking the calibration curve, and the mean absolute calibration error was only 0.006 (Figure 4E). Both Clinical impact curve and decision curve analysis (DCA) further affirm the clinical utility of the diagnostic nomogram (Figure 4C, 4F). Overall, the twelve HP-related hub genes could be expected to develop into the ideal diagnostic markers of GC.

**Figure 5:**
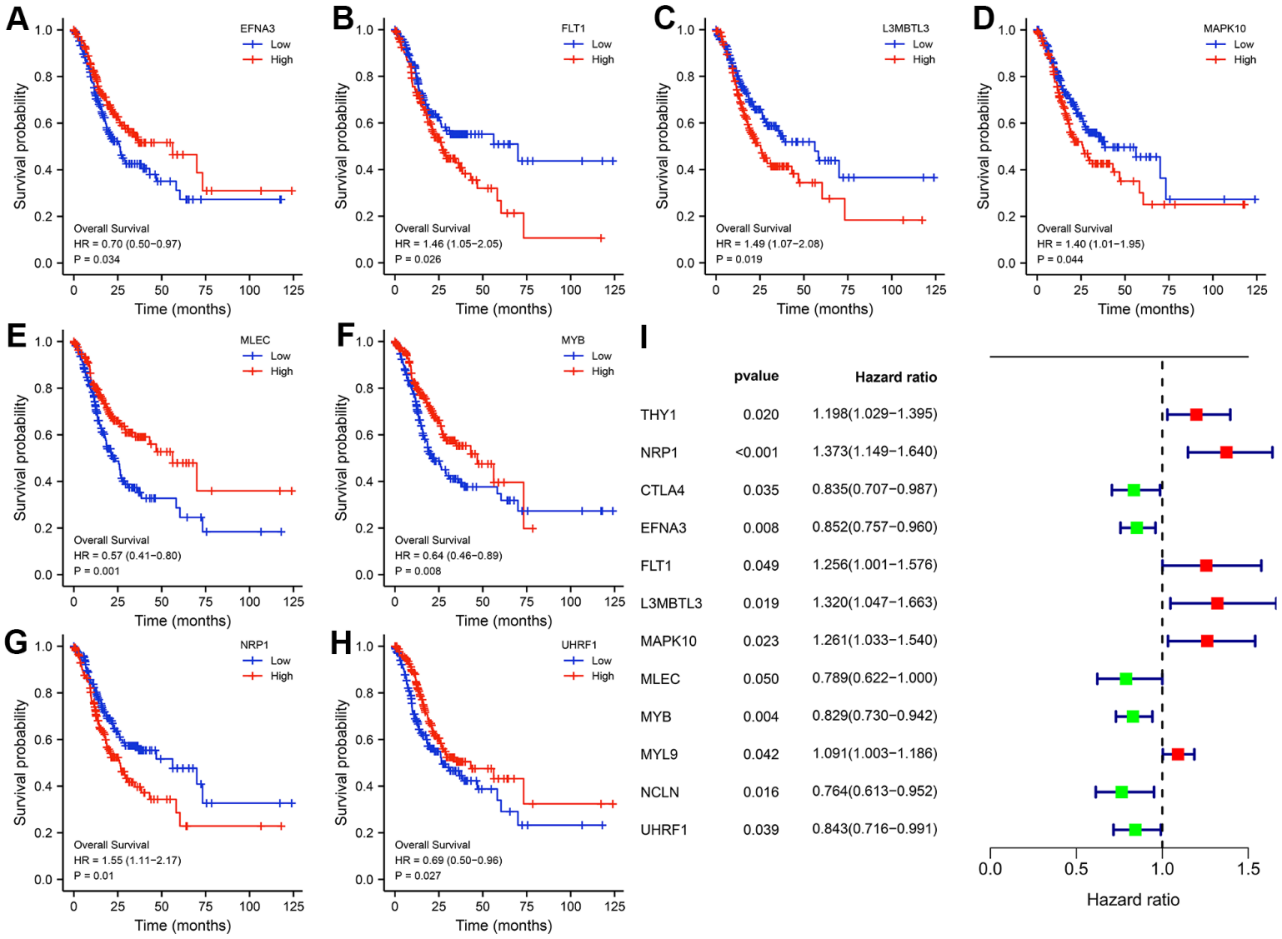
**Kaplan-Meier (K-M) survival curves of the hub genes.** (**A**) EFNA3. (**B**) FLT1. (**C**) L3MBTL3. (**D**) MAPK10. (**E**) MLEC. (**F**) MYB. (**G**) NRP1. (**H**) UHRF1. Univariate Cox regression analysis of the twelve hub genes. (**I**) Forest plot showing the prognostic values of hub genes.

Given that patient prognosis is what counts, we sorted all TCGA-STAD samples into high- or low -expression subgroups according to the median expression of the twelve hub genes for subsequent survival analysis. K-M curves uncovered that only eight hub genes (EFNA3, FLT1, L3MBTL3, MAPK10, MLEC, MYB, NRP1, as well as UHRF1) existed a significant difference in the clinical outcomes of GC patients between high and low-expression subgroups (Figure 5A–5H). However, univariate Cox regression analysis was also conducted to examine the association between the expression levels of hub genes and GC prognosis, and subsequent results revealed that all twelve hub genes were closely related to the survival time and status of patients with GC (Figure 5I). Thus, we could speculate boldly that the twelve HP-related hub genes affected these survival outcomes of GC patients together rather than individually.

Aside from this, the K-M Plotter database including six GEO datasets (GSE14210(N=145), GSE15459(N=200), GSE22377(N=43), GSE29272(N=268), GSE51105(N=94), GSE62254(N=300)) were also utilized to mine the prognostic value of hub genes. The results of KM Plotter website suggested that the expression levels of twelve hub genes exert a drastic effect on the survival time in GC patients whether it was OS or progression-free survival (PFS) (supplementary Figures 5, 6).

### Immune infiltration landscape of the hub genes

**Figure 6:**
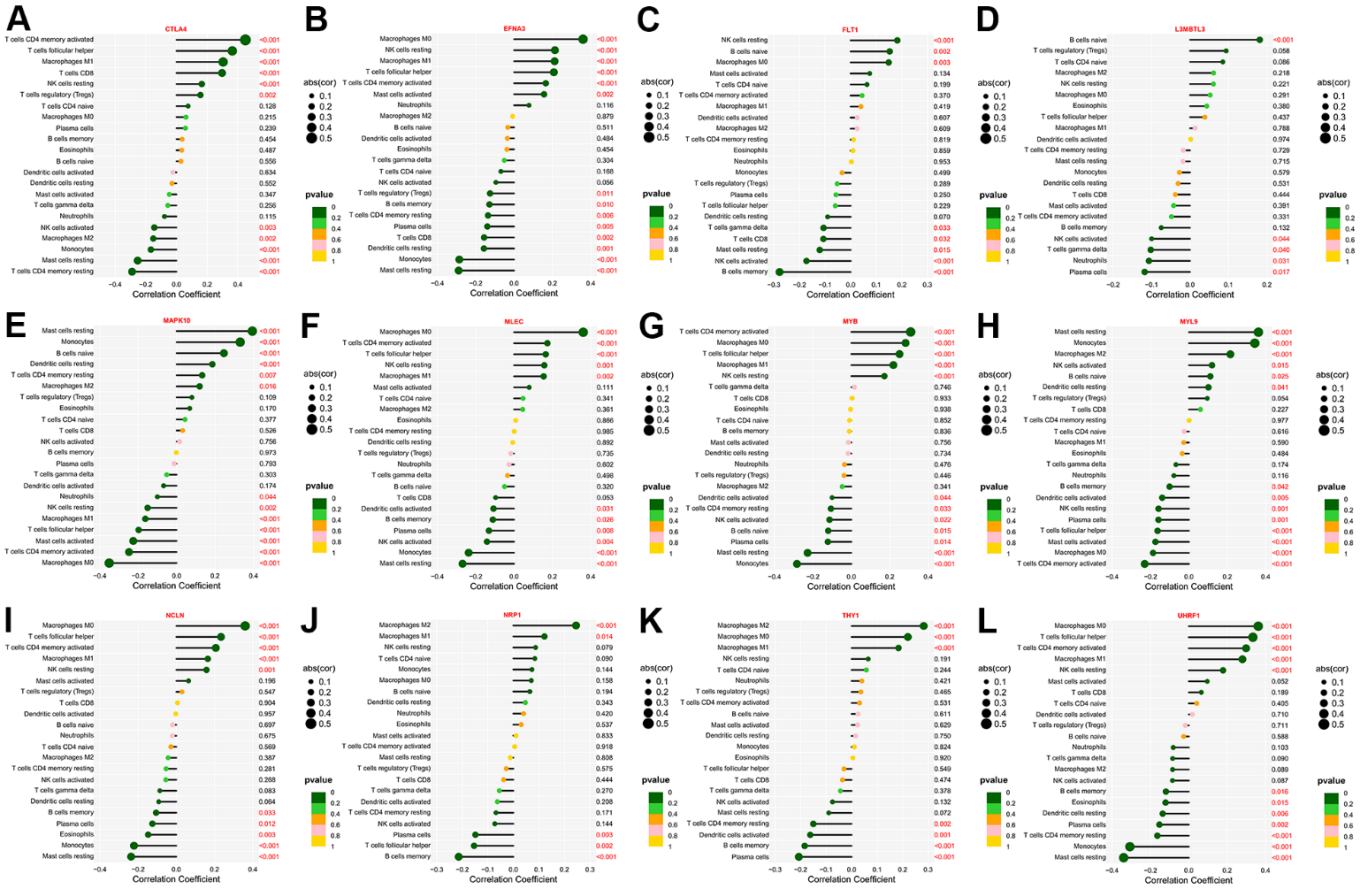
**The immune-infiltrating landscape of GC based on the twelve hub genes.** (**A**–**L**) Lollipop plots revealing the association between the twelve hub genes and the infiltration level of various immune cells.

The state of TME profoundly influences the efficacy of immunotherapy [32]. Using the CIBERSORT algorithm, we deconvoluted the composition ratio of distinct immune cell subpopulations in the TCGA- STAD patients. Subsequently, Spearman’s correlation analysis was conducted to discover the relationship between the expression levels of the twelve genes and the degree of immune cell infiltration. Expression of CTLA4 and MYB were negatively correlated with the infiltration abundance of Monocytes, Mast cells resting, as well as T cells CD4 memory resting, while presenting the highest positive correlation coefficient with T cells CD4 memory activated (Figure 6A, 6G). The expression of FLT1 was positively correlated with NK cells resting and B cells naive and negatively correlated with NK cells activated and B cells memory, while the expression level of L3MBTL3 was positively correlated with B cells naïve and Tregs and negatively correlated with Neutrophils and Plasma cells (Figure 6C, 6D). Similarly, NRP1 and THY1 were positively related to Macrophages M2 and Macrophages M1, whereas inversely related to B cells memory and Plasma cells (Figure 6J, 6K). Of interest, EFNA3, MLEC, NCLN together with UHRF1 all exhibited the highest positive correlation with Macrophages M0, and the most significant negative correlation with Mast cells resting (Figure 6B, 6F, 6I, 6L). Besides that, the expression levels of both MAPK10 and MYL9 were positively correlated with Mast cells resting, Monocytes, and B cells naive, but negatively correlated with T cells CD4 memory activated as well as Macrophages M0 (Figure 6E, 6H). Taken together, the hub genes could reshape the immune microenvironment to facilitate the initiation and progression of GC by altering the degree of various immune cell infiltration.

### Mutational characteristic of the hub genes

**Figure 7:**
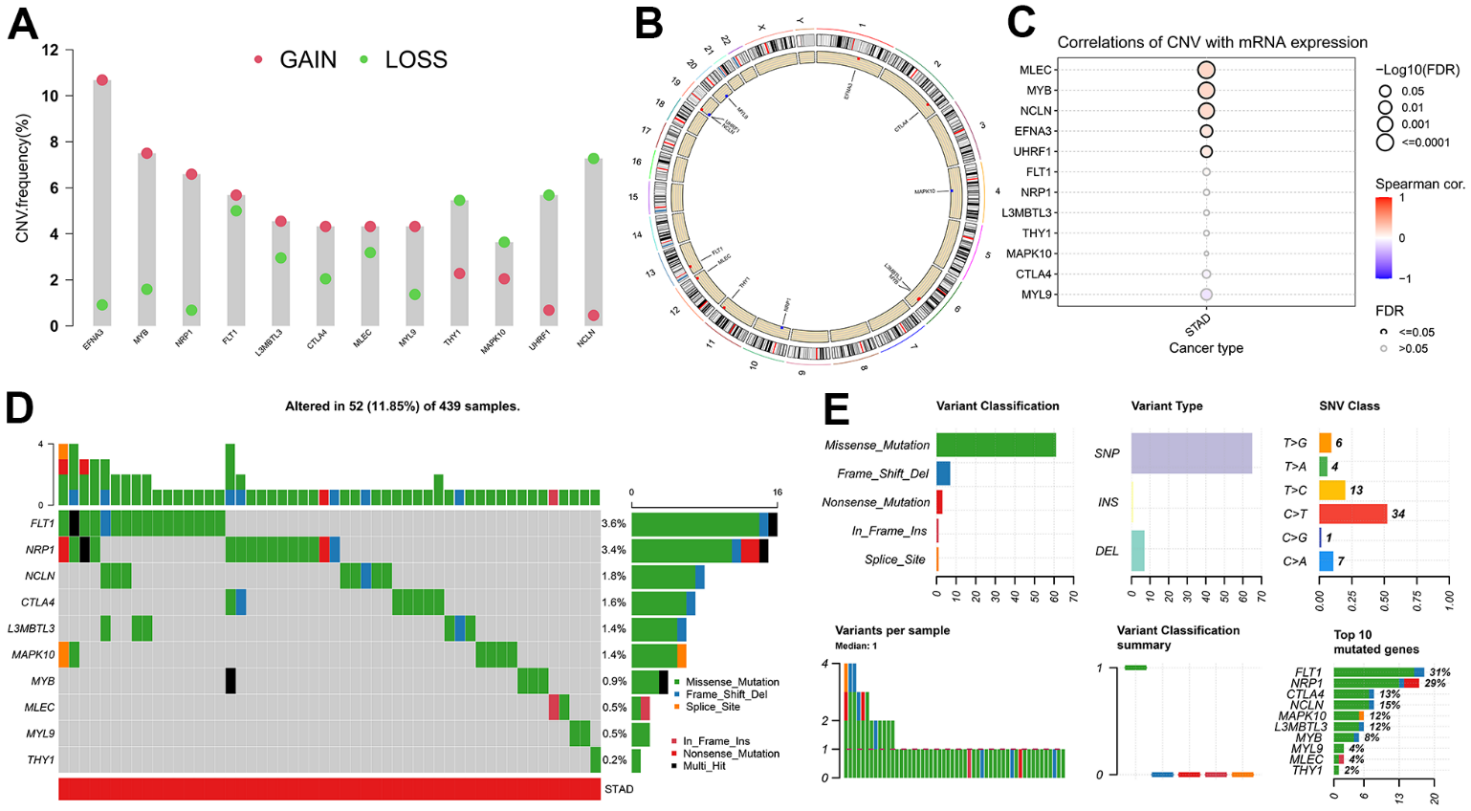
**Mutational characteristics of the hub genes.** (**A**) Copy number variation (CNV) frequency of hub genes. (**B**) Circle diagram of CNV with hub genes. (**C**) Correlation between expression of hub genes and CNV. (**D**) Cascade of hub gene mutations. (**E**) Details regarding single nucleotide variants (SNV).

As it is well-known, tumor mutation burden (TMB) has been increasingly recognized as being significantly associated with patient prognosis and immunotherapy response [33–35]. By exploiting the genomic alteration data from the TCGA database, the copy number variant (CNV) and single-nucleotide variant (SNV) events of the twelve hub genes were investigated to explore the relationship between expression and genetic mutation. EFNA3 exhibited the highest frequency of CNV, observed in exceeding 10% of TCGA-STAD samples, with CNV Gain being the more prevalent type compared to CNV Loss (Figure 7A). At the same time, the CNV frequencies over 3% for all hub genes were also found (Figure 7A). As displayed in Figure 7B, the chromosomal region and CNV state of all twelve genes were carefully marked on the schematic diagram. Further analysis suggested that the mRNA expression levels of MLEC, MYB, NCLN, EFNA3, and UHRF1 were positively related to their CNV frequencies (Figure 7C). Of the 439 GC samples, 52 (11.85%) occurred the alteration of hub genes, and FLT1 presented the highest SNV frequency (3.6%), followed by NRP1 (3.4%), NCLN (1.8%) (Figure 7D). Alongside this, subsequent results indicated that missense mutation (>60%), single-nucleotide polymorphism (SNP) (>60%), as well as C > T (34) were the most frequent classifications of SNV (Figure 7E).

In summary, among these 12 hub genes, the abnormal expression of EFNA3 and UHRF1 in GC compared with normal gastric tissues might be impacted by the regulation of CNV, whereas SNV was likely to affect the expression levels of FLT1, NRP1, CTLA4, L3MBTL3, MAPK10, MLEC, MYL9, as well as THY1, and MYB along with NCLN were perhaps subject to the joint effects of CNV and SNV.

### Assessment of drug sensitivity or resistance targeting the hub genes

**Figure 8:**
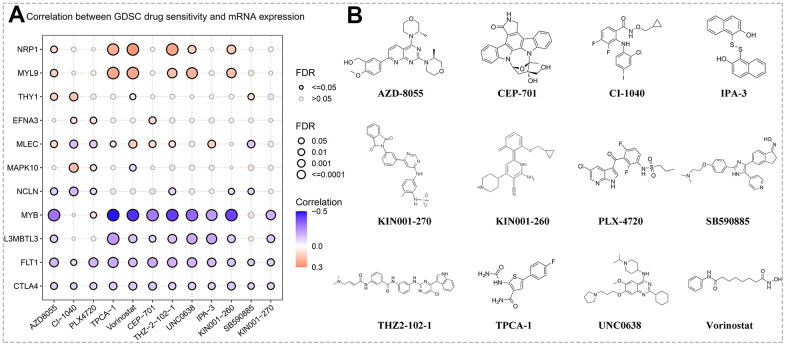
**Prediction of drug sensitivity.** (**A**) Correlation between hub gene expression levels and GSDC drug sensitivity via the online search tool GSCA. (**B**) Structural formulas of the sensitive agents (including AZD8055, CI-1040, PLX4720, TPCA-1, Vorinostat, CEP-701, THZ-2-102-1, UNC0638, IPA-3, KIN001-260, SB590885, and KIN001-270).

Chemotherapy and targeted therapy are still as important as surgery in the treatment of GC. Based on the GSCA database, we evaluated the association between the expression levels of hub genes and GDSC drug susceptibility or resistance. Subsequent results revealed that the sensitivity of several GDSC agents was positively and negatively correlated with the expression of multiple hub genes, including AZD8055, CI-1040, PLX4720, TPCA-1, Vorinostat, CEP-701, THZ-2-102-1, UNC0638, IPA-3, KIN001-260, SB590885, and KIN001-270 (Figure 8A). Moreover, the chemical and molecular structural formulas of the abovementioned twelve drugs were acquired by querying the MedChemExpress online website. The chemical formulas of AZD8055, CI-1040, PLX4720, TPCA-1, Vorinostat, CEP-701, THZ-2-102-1, UNC0638, IPA-3, KIN001-260, SB590885, and KIN001-270 corresponded to C_25_H_31_N_5_O_4_, C_17_H_14_CIF_2_IN_2_O_2_, C_17_H_14_CIF_2_N_3_O_3_S, C_12_H_10_FN_3_O_2_S, C_14_H_20_N_2_O_3_, C_26_H_21_N_3_O_4,_ C_31_H_28_CIN_7_O_2,_ C_30_H_47_N_5_O_2,_ C_20_H_14_O_2_S_2_, C_21_H_24_N_4_O_2,_ C_27_H_27_N_5_O_2,_ C_26_H_21_N_5_O_4_S_,_ separately. As shown in Figure 8B, the construct formulas of these agents were neatly arranged.

### Functional enrichment analysis

**Figure 9:**
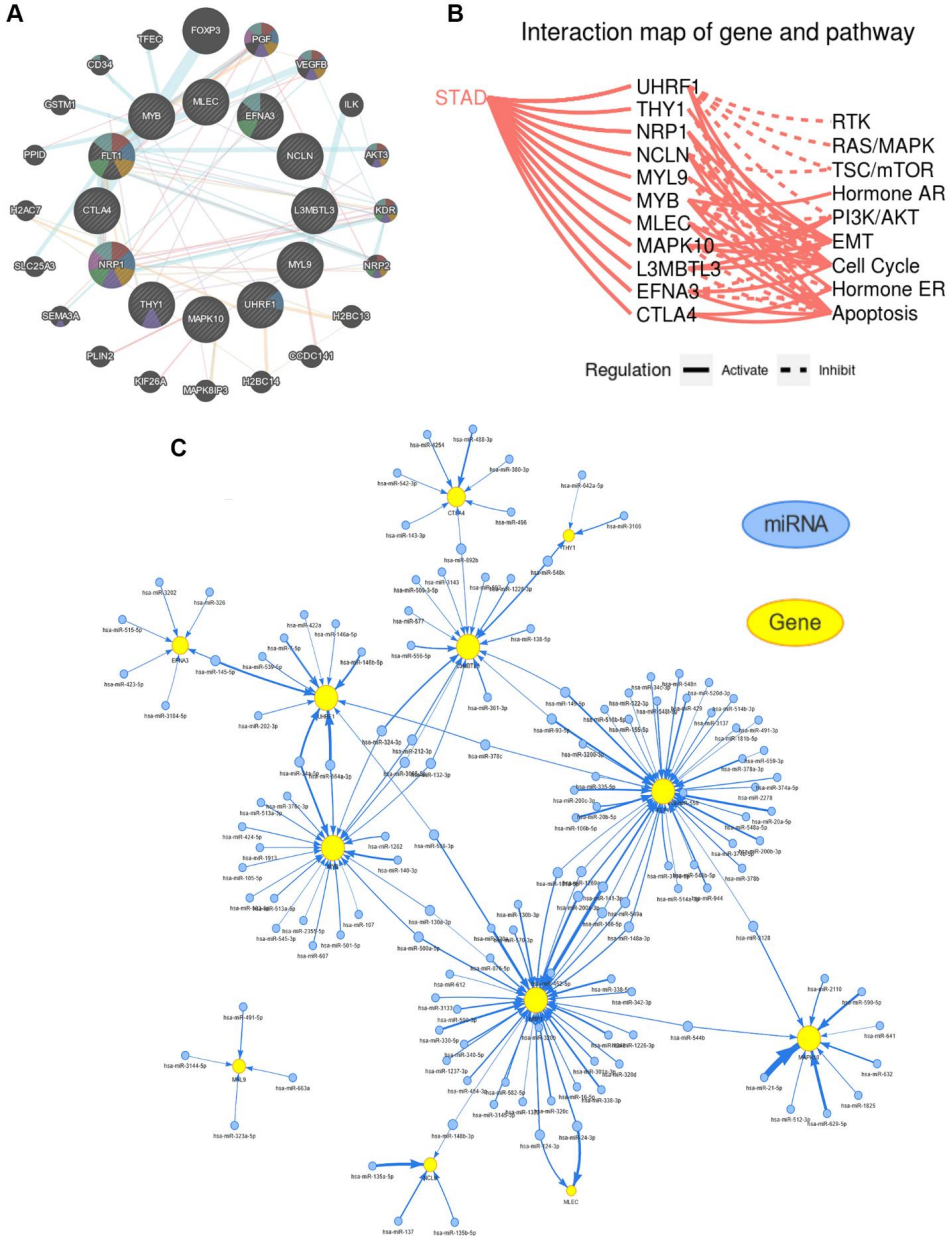
**Functional and pathway enrichment analysis of the hub genes.** (**A**) Construction of a protein-protein interaction (PPI) network through using the GeneMANIA database. (**B**) The hub genes being involved in several key cancer-associated processes, such as epithelial-mesenchymal transition (EMT), receptor tyrosine kinase (RTK), cell cycle, apoptosis, etc. (**C**) The result of predicted miRNAs targeting hub genes using the GSCALite website.

The synergy among hub genes was fully reflected in the above content. Therefore, to confirm the degree of tight junction of these hub genes, a PPI network was construed by using the GeneMANIA website (Figure 9A). To further examine the role of these hub genes in the occurrence and development of GC, the GSCALite database was utilized to investigate the interaction between hub genes and cancer-related pathways. The interaction maps uncovered that almost all hub genes were involved in the cancer-associated pathways, comprising RAS/MAPK, epithelial–mesenchymal transition (EMT), TSC/mTOR, receptor tyrosine kinase (RTK) signaling, hormone AR signaling, cell cycle, PI3K/AKT, hormone ER signaling, as well as apoptosis (Figure 9B). The upstream miRNAs is essential for understanding the oncogenic or tumor suppressor mechanism of gene, and a miRNA-mRNA regulatory network for hub genes was also created by using the GSCALite database, indicating that these hub genes shared common miRNAs to participate in various biological behavior of GC (Figure 9C). Furthermore, we again split TCGA-STAD samples into two subgroups (high- or low- expression) in accordance with the median expression of these hub genes for performing the GSEA analysis (Supplementary Figure 7). GSEA results showed that CTLA4, L3MBTL3, MAPK10, MLEC, MYB, MYL9, NCLN, NRP1, THY1, and UHRF1 were mainly enriched in pathways correlated with pathways in cancer, cell cycle, DNA replication, ECM-receptor interaction, cell adhesion molecules (CAMs), bladder cancer, renal cell carcinoma, focal adhesion, natural killer cell mediated cytotoxicity, T cell receptor signaling pathway, spliceosome, calcium signaling pathway, base excision repair, purine metabolism, pyrimidine metabolism, chemokine signaling pathway, mTOR signaling pathway, N-glycan biosynthesis, in the high-expression subgroup, whereas EFNA3 and FLT1 were involved in the calcium signaling pathway, ribosome, oxidative phosphorylation, hematopoietic cell lineage, Parkinsons disease, and vascular smooth muscle contraction in the low-expression subgroup.

### Validation of differential expression for hub genes

**Figure 10:**
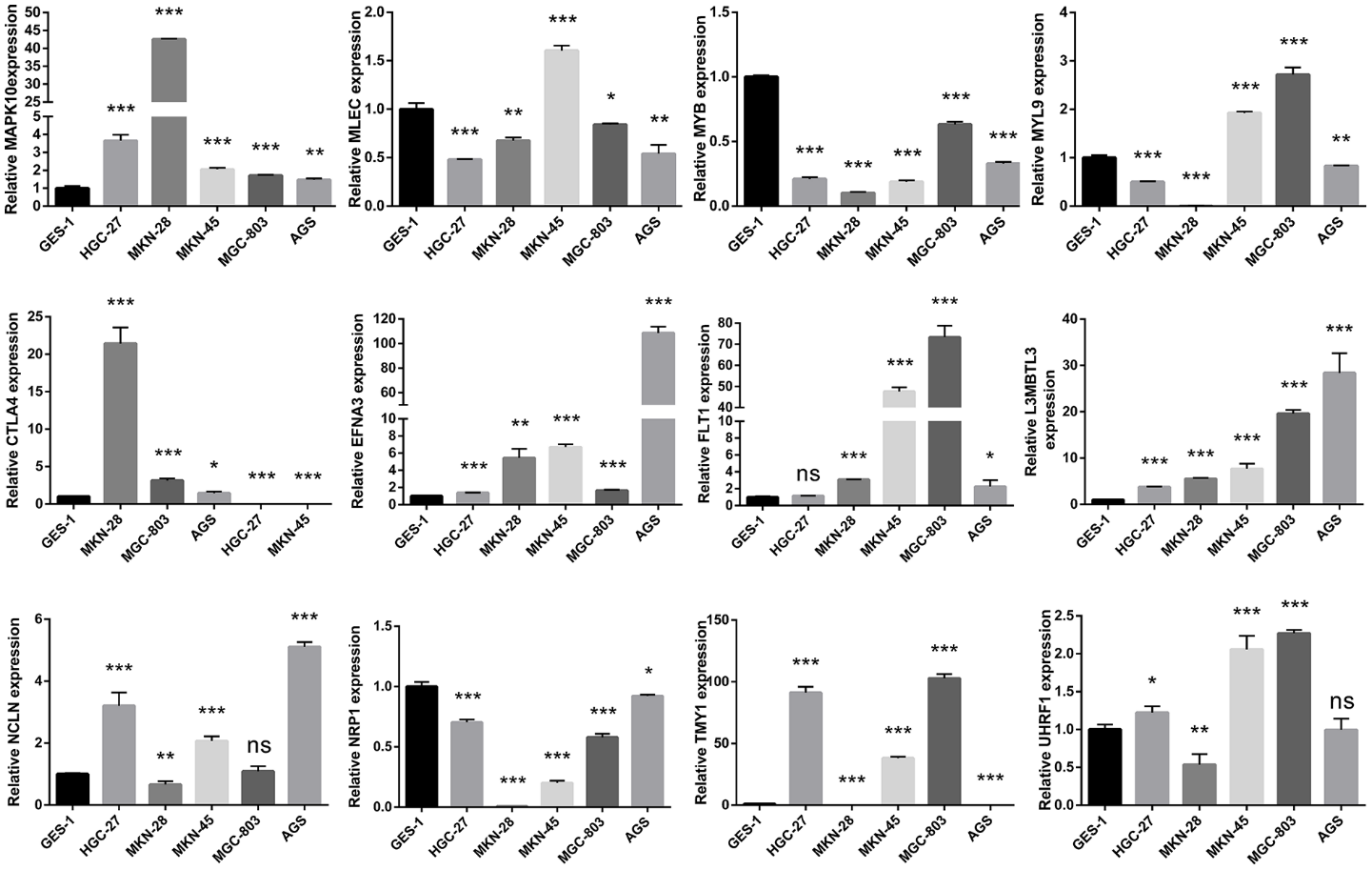
Validating the mRNA expression of the twelve hub genes in normal gastric epithelial and GC cell lines via the quantitative reverse transcription polymerase chain reaction (qRT-PCR) assays.

The qRT-PCR assay was commonly applied to measure the mRNA expression level of gene, and we compared the mRNA expression of hub genes in normal gastric epithelial and multiple GC cells through the qRT-PCR experiment in this present study, suggesting that all twelve of these genes were obviously different (Figure 10). The HPA website is a repository of immunohistochemistry-based proteomic data that provides us with significant value for protein expression analysis [36]. The IHC staining image were retrieved and downloaded from the HPA database to validate the protein expression of hub genes, indicating that these genes were differentially expressed between GC and adjacent normal tissues, and the trend of up-regulation or down-regulation was basically consistent with the mRNA expression level (Supplementary Figure 8 and Supplementary Table 7).

## DISCUSSION

It is well known that HP infection is a premalignant form of GC and that it plays a key role in the development and spread of the disease [37]. Although eliminating HP can greatly lower the frequency of GC, its prevalence is still high, particularly in underdeveloped nations [38–40]. The relationship between HP infection and GC has been the subject of numerous studies, but these studies primarily concentrate on the oncogenic effects of HP strains’ virulence factors, such as *BabA*, *CagA*, *oipA*, and *VacA*, as well as environmental risk factors like increased or decreased gastric fluid pH and nitrosamines and their precursors [41–44]. On the molecular and genetic levels, nevertheless, there are very few investigations on the tumorigenic processes of HP infection. It is generally established that dysregulation of oncogene and tumor-suppressor genes can contribute to the beginning and growth of tumors. Therefore, in this investigation, we sought to clarify the therapeutic and prognostic effects of HP infection on GC based on HP-related genes.

At the beginning of this present study, we conducted NMF clustering analysis on the TCGA-STAD, GSE15459, GSE84433 cohorts based on 232 differentially expressed HP-related genes. Subsequent results revealed that all different data sets’ samples were stratified into two distinct clusters with differing prognosis, immune infiltration landscape, and anticancer drug sensitivity, indicating that the HP-related genes could exert a significant influence on the clinical outcomes, tumor microenvironment, as well as therapeutic efficacy of GC.

To determine the most critical HP-related genes, two ML methods, SVM-RFE together with RF, were employed to identify the HP-associated hub genes. SVM-RFE, a quite well-established ML algorithm for classification, can achieve the selection of optimal features on the basis of the recursive feature elimination [45]. RF is also a supervised non-parametric ML approach, which can be applied to address classification and regression issues, including gene screening and disease diagnosis [46]. Twelve genes were eventually screened and identified as the HP-related hub genes by intersecting of the results of two ML strategies, namely, EFNA3, UHRF1, FLT1, NRP1, CTLA4, L3MBTL3, MAPK10, MLEC, MYL9, THY1, MYB, as well as NCLN, since both the SVM-RFE and RF models presented less residuals and higher ROC values.

It has previously been demonstrated that multiple hub genes are closely implicated in tumorigenesis and progression. For instance, EFNA3/EPHA2 axis can promote cancer stemness in hypoxic hepatocellular carcinoma by modulating metabolic plasticity, and EFNA3 is served as a prognostic biomarker for hepatocellular cancer [47, 48]. UHRF1 can facilitate the occurrence and development of various digestive tract tumors, including gastric, colon, and pancreatic cancers, etc. [49–51]. FLT1 and its ligands VEGFB together with PlGF are promising as key targets for a new generation of anti-angiogenic drugs [52]. NRP1 is closely associated with the occurrence, progression and even metastasis of various tumors, such as bladder, colorectal, breast, and lung cancers [53–56]. CTLA-4 has been generally recognized as the most compelling target immunotherapy, and ipilimumab (anti-CTLA4) has radically and significantly improved the clinical outcomes of patients with advanced cancer [57]. Dysregulated miR-27a-3p enhances the proliferation and migration capability of nasopharyngeal carcinoma cell by regulating the expression level of MAPK10, and Circ_0000515 can also drive hepatocellular carcinoma progression by targeting MAPK10 [58, 59]. Aberrant Expression of MYL9 is correlated with prognosis of glioblastoma and esophageal squamous cell cancer, and it may act as a novel biomarker [60, 61]. MicroRNA-140-5p suppresses the growth and progression of GC cells by reversely modulating THY1-mediated Notch signaling [62]. SNHG3 promotes the proliferation and metastatic ability of GC cells by mediating the miR-139-5p/MYB axis [63].

To examine the diagnostic performance of the twelve hub genes, ROC curves of each hub genes were plotted and their AUC values were calculated. All hub genes reached high AUC values, with UHRF1 exhibiting the highest AUC value of 0.958. To predict the likelihood of initiation of GC, a nomogram on the basis of the twelve HP-related hub genes was constructed. To illustrate the clinical significance of the hub genes, we performed survival analysis (K-M and univariate Cox regression analyses). Subsequent results suggested that the expression levels of the nine hub genes was closely to the survival outcomes of patients with GC in both TCGA and GEO cohorts.

Immunotherapy has emerged as a promising treatment strategy for GC, yet drug sensitivity varies from person to person. The composition of immune cell infiltration affects the immunotherapy response and is served as a significant determinant [64]. In this research, expression of CTLA4, MYB, FLT1, L3MBTL3, MAPK10 and MYL9 positively correlated with the abundance of antitumor immune cells (e.g., T cells CD4, Mast cells, NK cells, B cells, etc.), while NRP1 and THY1 presented a negative association with the infiltration level of immunosuppressive Macrophages M2. TMB is another indicator for evaluating the response to immunotherapy [65]. In the TCGA-STAD dataset, EFNA3 showed the highest frequency of CNV, found in more than 10% of patients, with CNV Gain being the more prevalent type. At the same time, FLT1 exhibited the highest SNV frequency (3.6%), with missense mutation and C > T being the main classification.

At final, we also discovered that these hub genes may participate in the onset and progression of GC via the following cancer-associated pathways, namely, cell cycle, EMT, apoptosis, RAS/MAPK, PI3K/AKT, TSC/mTOR, hormone AR signaling, hormone ER signaling, as well as RTK. Besides that, the twelve hub genes’ upstream regulatory miRNAs were predicted.

## CONCLUSIONS

In this study, we developed a unique HP-related tumor classification and performed ML techniques to identify twelve hub genes that may be useful for GC molecular diagnosis and individualized treatment.

## Supplementary Material

Supplementary Figures

Supplementary Table 1

Supplementary Table 2

Supplementary Table 3

Supplementary Tables 4-6

Supplementary Table 7
