# Supplementary material for: Hub gene identification and molecular subtype construction for Helicobacter pylori in gastric cancer via machine learning methods and NMF algorithm
Source: Aging (Albany NY). 2023 Sep 26;15(21):11782–810. doi: 10.18632/aging.205053 (PMC10683617; doi:10.18632/aging.205053)
Supplement: Supplementary Figures [file aging-15-205053-s001.pdf]

SUPPLEMENTARY FIGURES

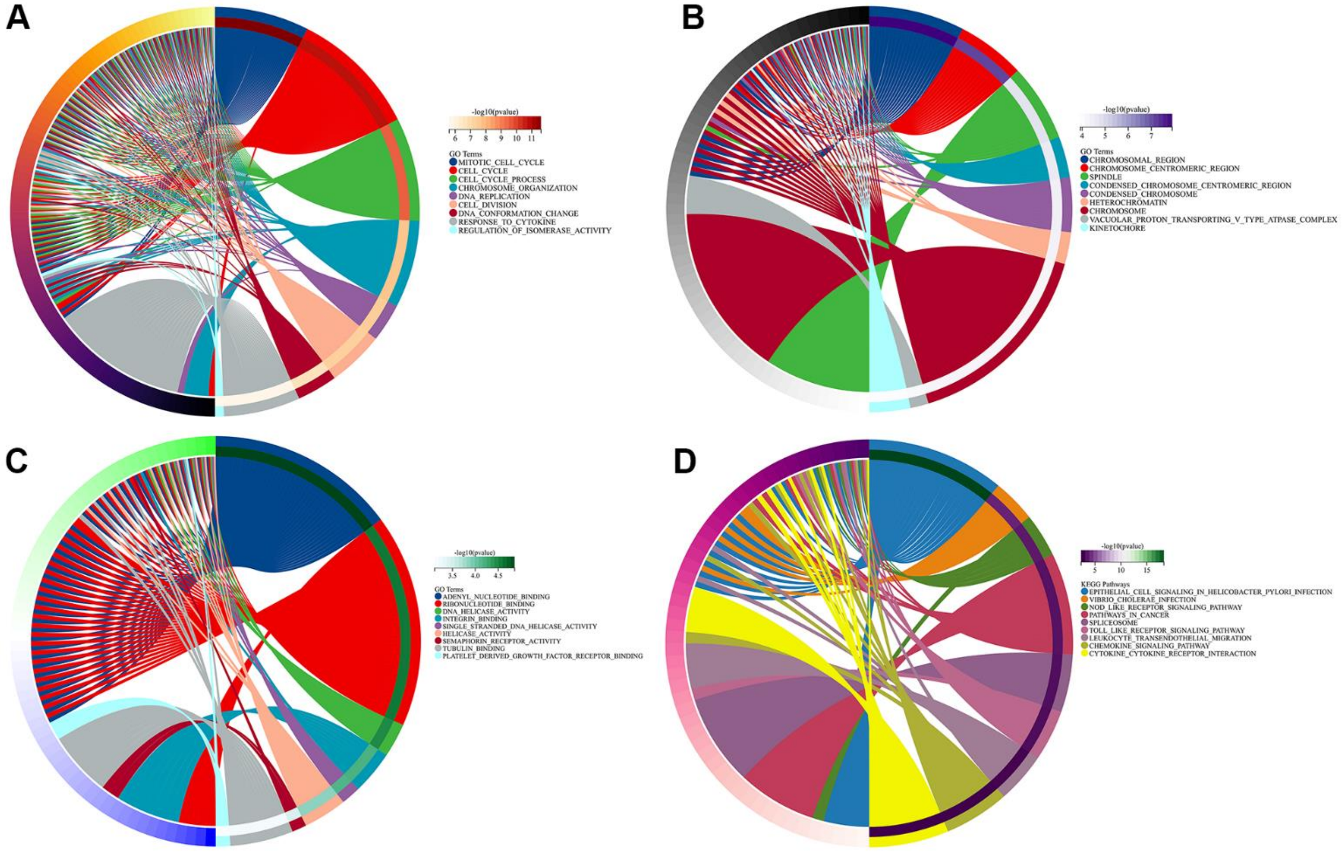

Supplementary Figure 1. Functional enrichment analysis (**A**–**C**) GO analysis (biological process (BP), cellular component (CC), and molecular function (MF)) of the differentially expressed HP-related genes. (**D**) KEGG analysis of the differentially expressed HP-related genes.

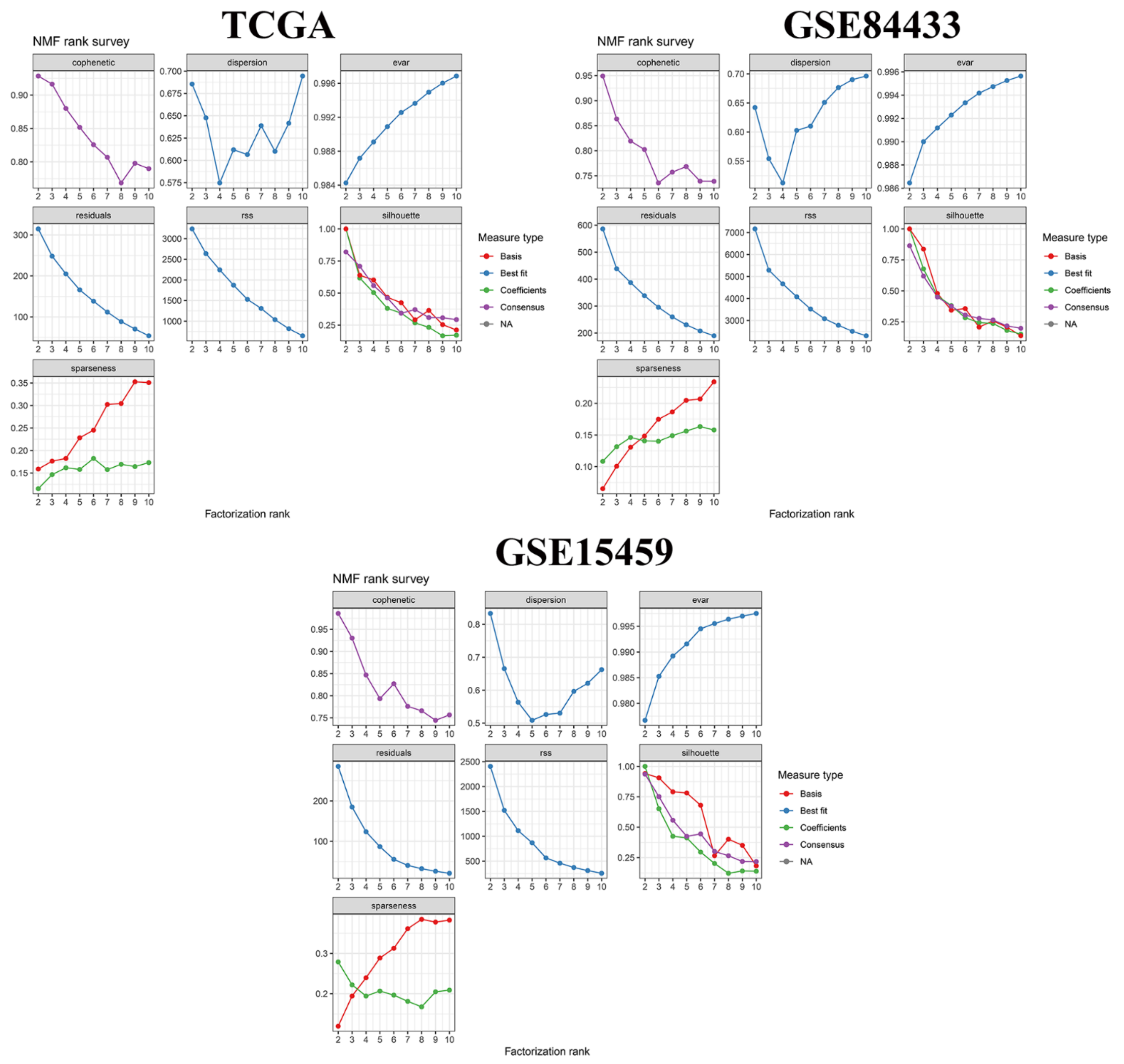

Supplementary Figure 2. NMF rank survey performed on distinct clusters in multiple datasets.

GSE84433 Dataset

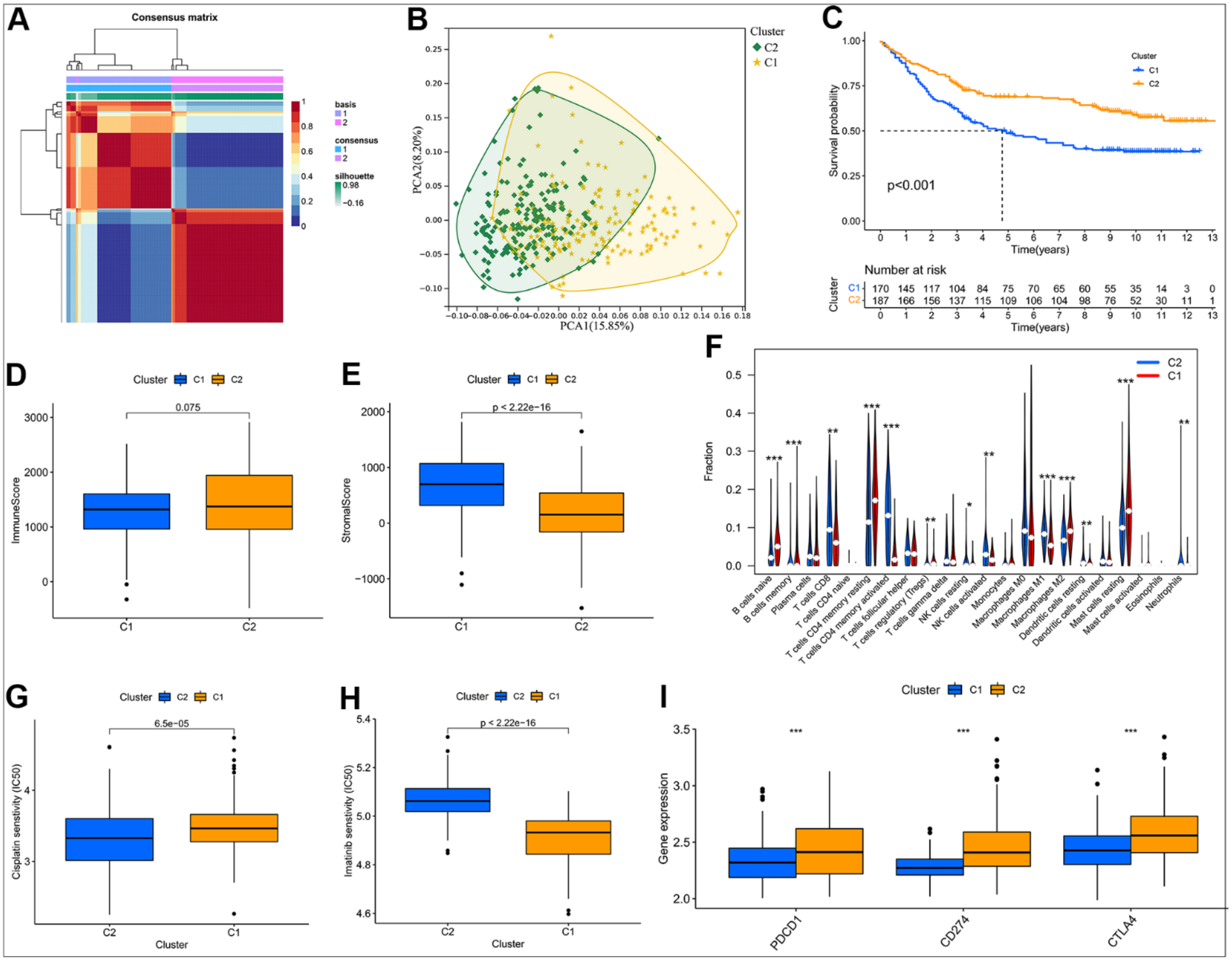

Supplementary Figure 3. Establishment of a NMF subtype according to the differentially expressed HP-related genes in the GSE84433 cohort. (**A**) NMF consensus clustering for k = 2. (**B**) Kaplan–Meier analysis of overall survival (OS) for Cluster C1 and C2. (**C**) Principal component analysis (PCA). (**D**, **E**) Differential analyses of immune and stromal score between Cluster C1 and C2. (**F**) Violin plot showing the immune cell infiltration landscape across different clusters. (**G**, **H**) Box plot of estimated IC50 values for Cisplatin and Imatinib in Cluster C1 and C2. (**I**) Box plot visualizing the significant expression differences of immune checkpoints across distinct clusters, including PDCD1, CD274, and CTLA4. *:P<0.05 ** :P<0.01 ***:P<0.001.

GSE15459 Dataset

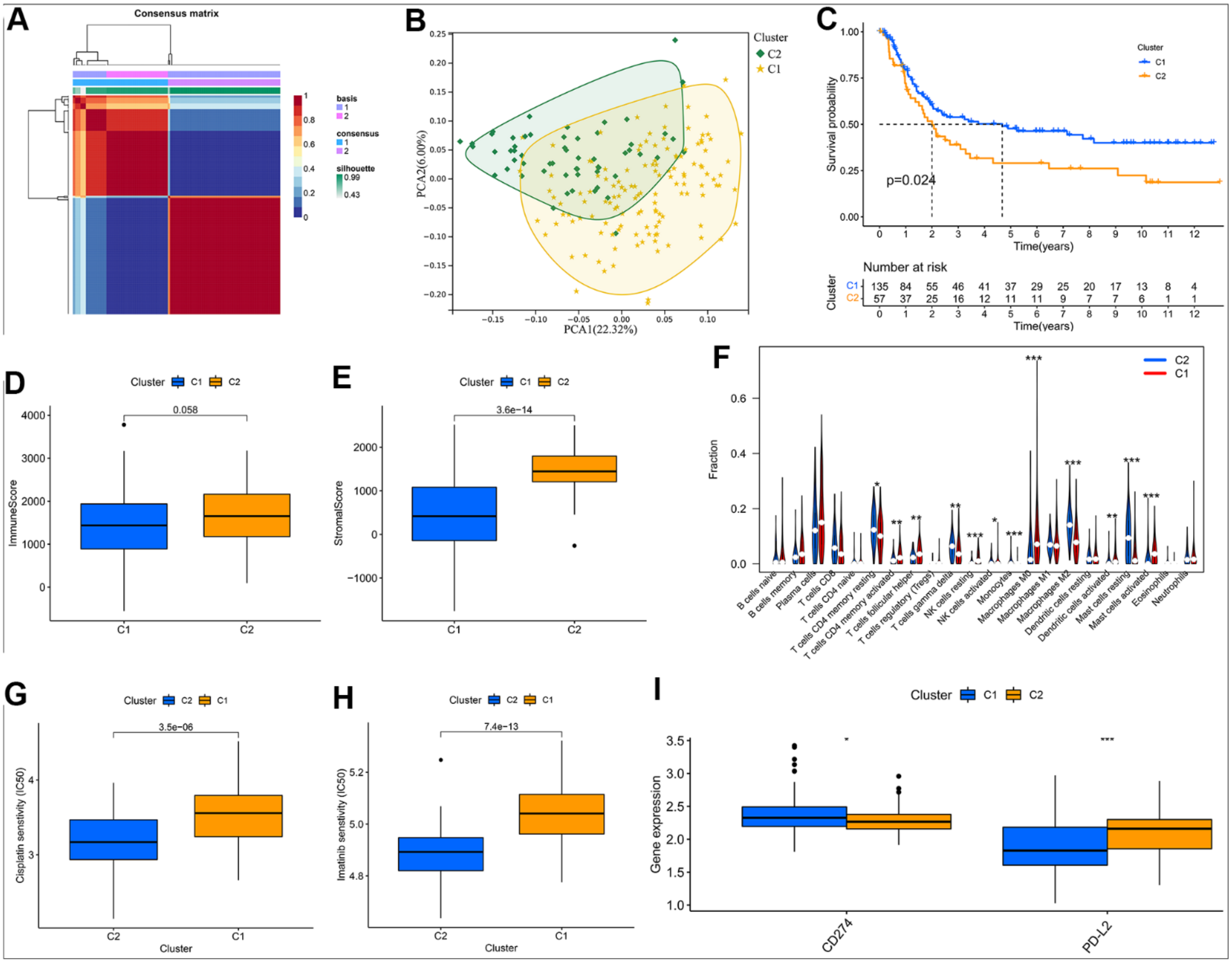

Supplementary Figure 4. Creation of a NMF subtype based on the differentially expressed HP-related genes in the GSE15459 cohort. (**A**) NMF consensus clustering for k = 2. (**B**) Kaplan–Meier analysis of overall survival (OS) for Cluster C1 and C2. (**C**) Principal component analysis (PCA). (**D**, **E**) Differential analyses of immune and stromal score between Cluster C1 and C2. (**F**) Violin plot showing the immune cell infiltration landscape across different clusters. (**G**, **H**) Box plot of estimated IC50 values for Cisplatin and Imatinib in Cluster C1 and C2. (**I**) Box plot visualizing the significant expression differences of immune checkpoints across distinct clusters, including CD274 and PD-L2. *:P<0.05 ** :P<0.01 ***:P<0.001.

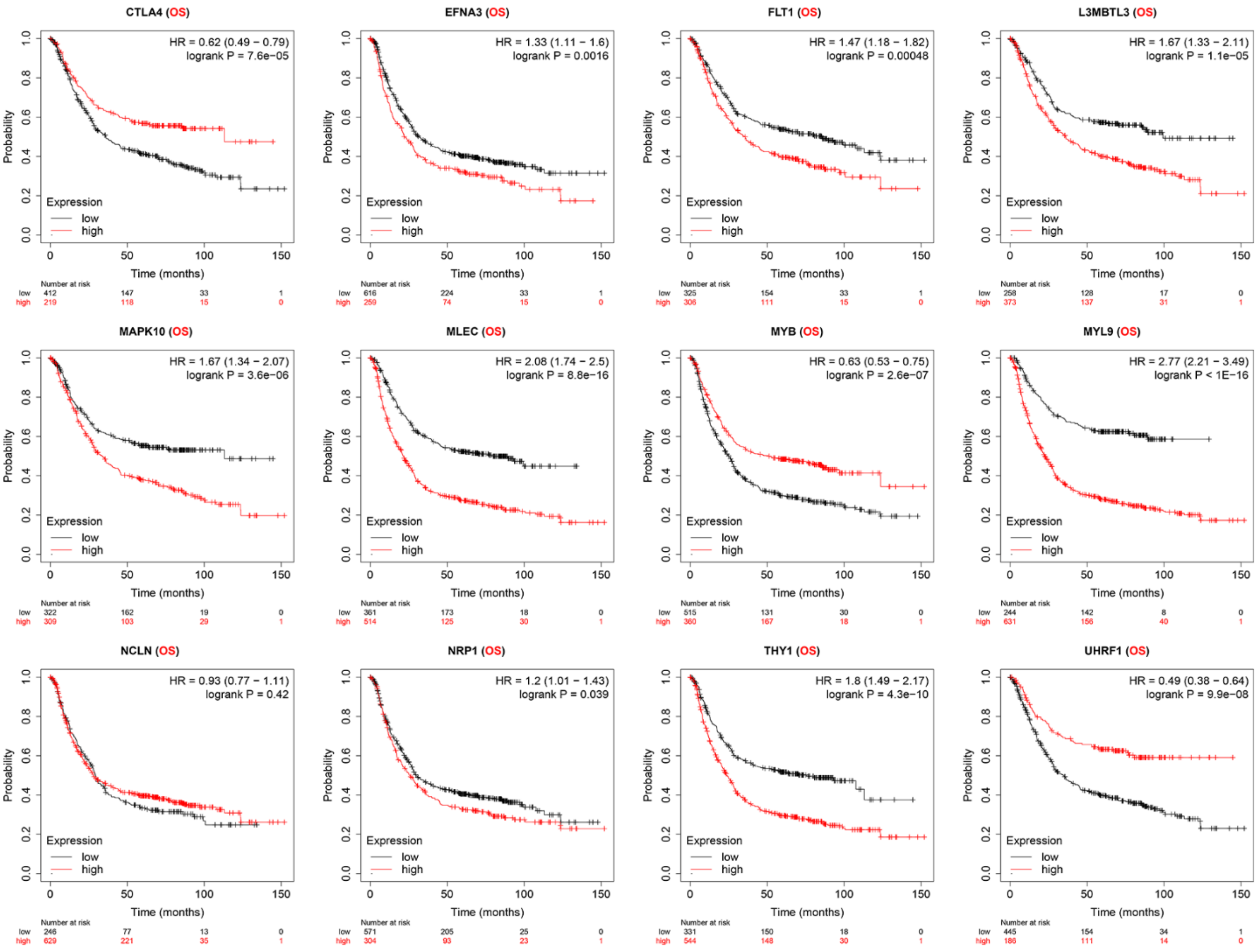

Supplementary Figure 5. Six GEO datasets confirming the prognosis value of hub genes (OS).

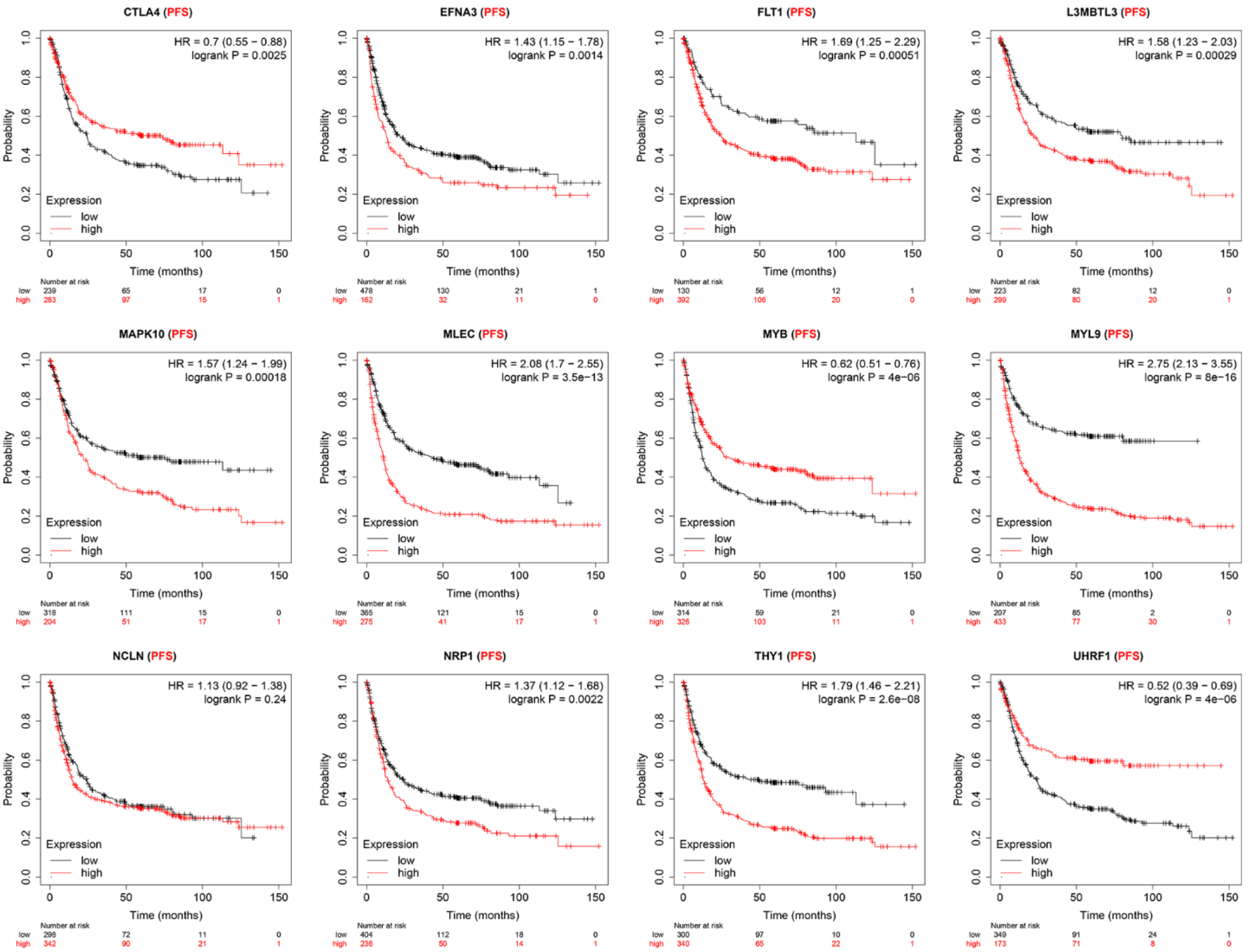

Supplementary Figure 6. Six GEO datasets confirming the prognosis value of hub genes (PFS).

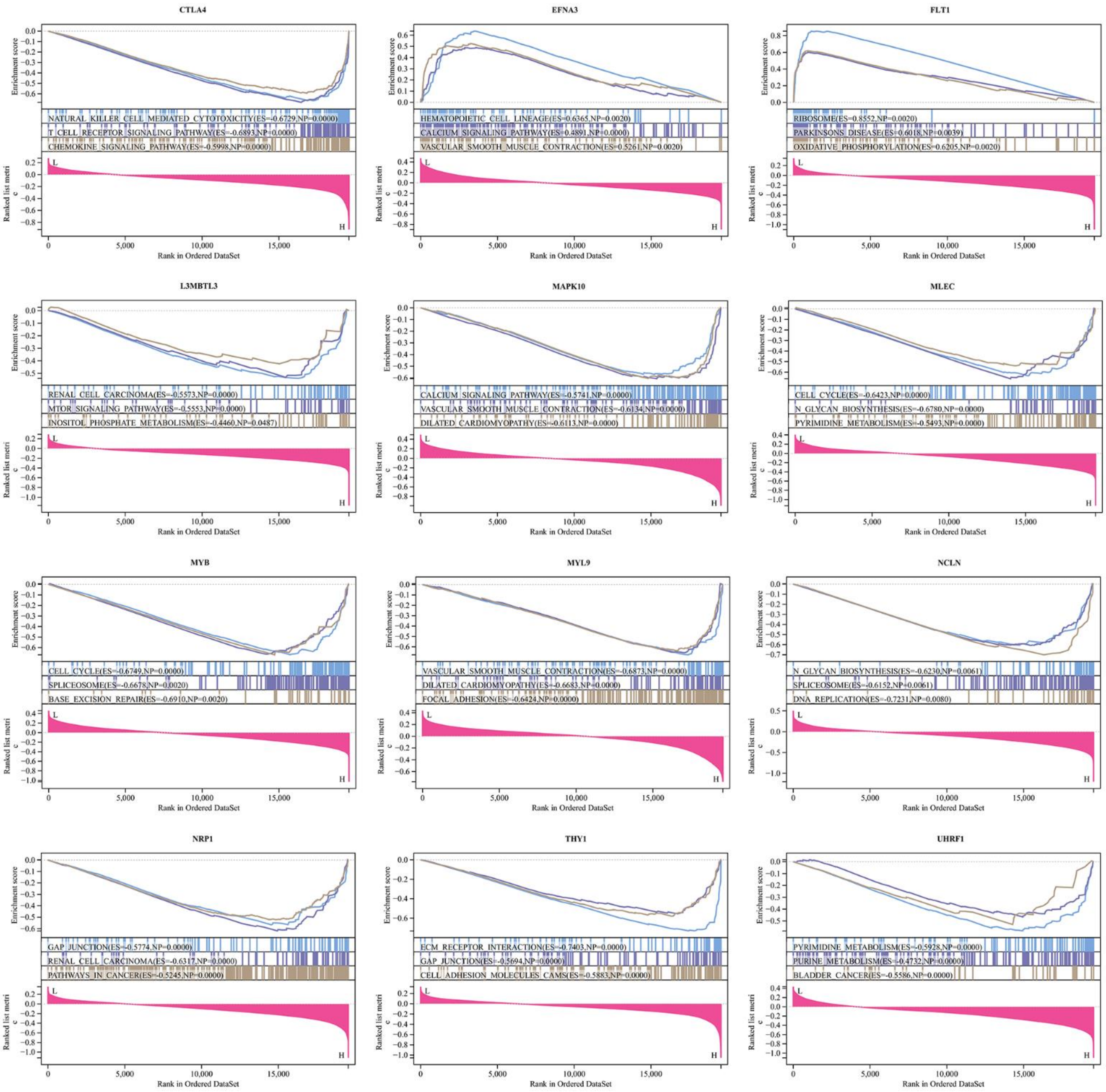

Supplementary Figure 7. Gene set enrichment analysis (GSEA) of each hub gene.

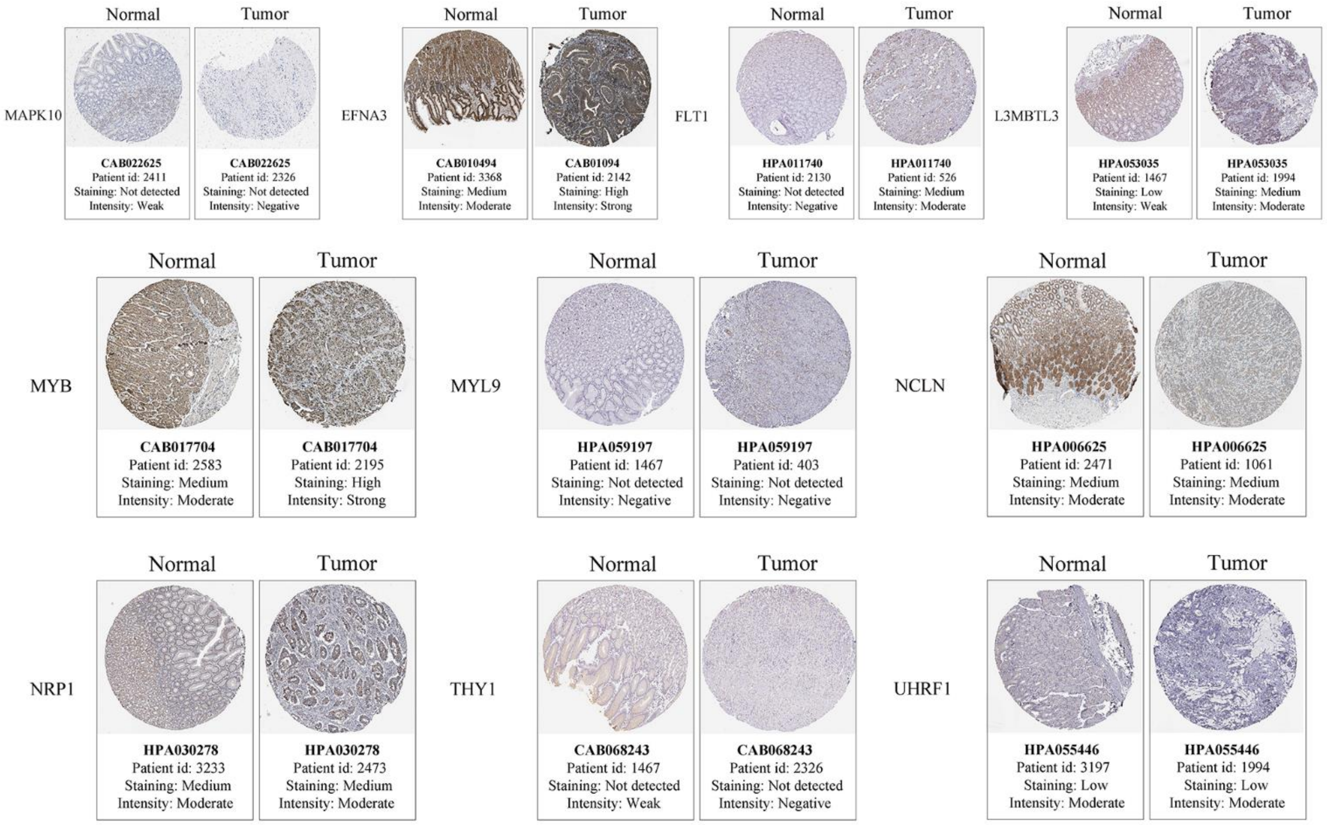

Supplementary Figure 8. HPA database verifying the protein expression of hub genes (EFNA3, UHRF1, FLT1, NRP1, L3MBTL3, MAPK10, MYL9, THY1, MYB, as well as NCLN).
