# Supplementary material for: Hub gene identification and molecular subtype construction for Helicobacter pylori in gastric cancer via machine learning methods and NMF algorithm
Source: Aging (Albany NY). 2023 Sep 26;15(21):11782–810. doi: 10.18632/aging.205053 (PMC10683617; doi:10.18632/aging.205053)
Supplement: Supplementary Table 1 [file aging-15-205053-s002.pdf]

SUPPLEMENTARY TABLES

Supplementary Table 1. Primers sequences in qRT-PCR.

Primers sequences	Forward sequence	Reverse sequence
GAPDH	CCCACTCCTCCACCTTTGAC	CCACCACCCTGTTGCTGTAG
EFNA3	AGTTCTCGGAGAAGTTCCAGCG	CAGCAGACGAACACCTTCATCC
FLT1	CCTGCAAGATTCAGGCACCTATG	GTTTCGCAGGAGGTATGGTGCT
L3MBTL3	TTCGCAGAGAGCACGGAGGAA	ACCGCTTTCTCCTCTTCCAGGT
MAPK10	GCACACACACATGCATACCC	TCTCACTGCTCAGACCTTGC
MLEC	GGGCAGGATGGGTATGCTTT	CGGTTCTGCTTCCGTGTACT
MYB	GGGAACAGATGGGCAGAAATCG	GCTGGCTTTTGAAGACTCCTGC
MYL9	GGATGTGATTCGCAACGCCTTTG	CGGTACATCTCGTCCACTTCCT
NCLN	ACCTCCTGTTCTTTGCGTCTGG	CCACATTGTCCTGAAGCAGGCT
NRP1	AACAACGGCTCGGACTGGAAGA	GGTAGATCCTGATGAATCGCGTG
THY1	CTCCAGCATTCTCAGCCACA	CGCTGCTTTCCTGGTCAAAC
UHRF1	GACAAGCAGCTCATGTGCGATG	AGTACCACCTCGCTGGCATCAT
CTLA4	ACGGGACTCTACATCTGCAAGG	GGAGGAAGTCAGAATCTGGGCA
