# Supplementary material for: Hub gene identification and molecular subtype construction for Helicobacter pylori in gastric cancer via machine learning methods and NMF algorithm
Source: Aging (Albany NY). 2023 Sep 26;15(21):11782–810. doi: 10.18632/aging.205053 (PMC10683617; doi:10.18632/aging.205053)
Supplement: Supplementary Tables 4-6 [file aging-15-205053-s005.pdf]

Supplementary Table 4. 17 prognostic-associated HP genes.

Id	HR	HR.95L	HR.95H	pvalue
THY1	1.197780366	1.028640775	1.394731611	0.020150799
NRP1	1.372799425	1.149153022	1.63997155	0.000478942
PLXNC1	1.198026544	1.012051655	1.418176228	0.035803952
NCLN	0.763501882	0.612545701	0.951659808	0.016359488
EFNA3	0.852098441	0.756579369	0.959676914	0.008328384
MYL9	1.090867971	1.003169012	1.186233741	0.041955934
CPT1C	1.193375145	1.026920507	1.386810592	0.021079546
TMEM176B	1.168858366	1.001461307	1.364236312	0.047874547
MYB	0.829498517	0.730244348	0.942243225	0.004041507
L3MBTL3	1.319766027	1.047446338	1.662884582	0.018617422
CTLA4	0.835480337	0.707075962	0.987202833	0.034751367
MAPK10	1.260993457	1.032619842	1.53987405	0.02291534
PDE2A	1.195255496	1.040593573	1.372904597	0.011642923
MLEC	0.788755252	0.622210985	0.999877633	0.04988191
FLT1	1.256261141	1.001183574	1.576326355	0.048817849
ANKRD33	1.247838303	1.04927556	1.483976651	0.012282632
UHRF1	0.842653628	0.716375984	0.991190593	0.038754336

Supplementary Table 5. Thirteen candidate genes were selected by using the random forest (RF) method.

Gene
UHRF1
THY1
CTLA4
MLEC
MAPK10
EFNA3
MYL9
NCLN
MYB
NRP1
L3MBTL3
CPT1C
FLT1

Supplementary Table 6. Thirteen genes were screened by establishing the support vector machine-recursive feature elimination (SVM-RFE) model.

Gene
UHRF1
EFNA3
MLEC
THY1
MYB
CTLA4
MAPK10
NCLN
MYL9
FLT1
NRP1
L3MBTL3
PDE2A
