# Supplementary material for: Hub gene identification and molecular subtype construction for Helicobacter pylori in gastric cancer via machine learning methods and NMF algorithm
Source: Aging (Albany NY). 2023 Sep 26;15(21):11782–810. doi: 10.18632/aging.205053 (PMC10683617; doi:10.18632/aging.205053)
Supplement: Supplementary Table 7 [file aging-15-205053-s006.docx]

Supplementary Table 7. The direct link URLs to the cited images.

MAPK10-Normal

<https://www.proteinatlas.org/ENSG00000109339MAPK10/tissue/stomach#img>

MAPK10-Tumor

<https://www.proteinatlas.org/ENSG00000109339-MAPK10/pathology/stomach+cancer#img>

EFNA3-Normal

<https://www.proteinatlas.org/ENSG00000143590-EFNA3/tissue/stomach#img>

EFNA3-Tumor

<https://www.proteinatlas.org/ENSG00000143590-EFNA3/pathology/stomach+cancer#img>

FLT1-Normal

<https://www.proteinatlas.org/ENSG00000102755-FLT1/tissue/stomach#img>

FLT1-Tumor

<https://www.proteinatlas.org/ENSG00000102755-FLT1/pathology/stomach+cancer#img>

L3MBTL3-Normal

<https://www.proteinatlas.org/ENSG00000198945-L3MBTL3/tissue/stomach#img>

L3MBTL3-Tumor

<https://www.proteinatlas.org/ENSG00000198945-L3MBTL3/pathology/stomach+cancer#img>

MYB-Normal

<https://www.proteinatlas.org/ENSG00000118513-MYB/tissue/stomach#img>

MYB-Tumor

<https://www.proteinatlas.org/ENSG00000118513-MYB/pathology/stomach+cancer#img>

MYL9-Normal

<https://www.proteinatlas.org/ENSG00000101335-MYL9/tissue/stomach#img>

MYL9-Tumor

<https://www.proteinatlas.org/ENSG00000101335-MYL9/pathology/stomach+cancer#img>

NCLN-Normal

<https://www.proteinatlas.org/ENSG00000125912-NCLN/tissue/stomach#img>

NCLN-Tumor

<https://www.proteinatlas.org/ENSG00000125912-NCLN/pathology/stomach+cancer#img>

NRP1-Normal

<https://www.proteinatlas.org/ENSG00000099250-NRP1/tissue/stomach#img>

NRP1-Tumor

<https://www.proteinatlas.org/ENSG00000099250-NRP1/pathology/stomach+cancer#img>

THY1-Normal

<https://www.proteinatlas.org/ENSG00000154096-THY1/tissue/stomach#img>

THY1-Tumor

<https://www.proteinatlas.org/ENSG00000154096-THY1/pathology/stomach+cancer#img>

UHRF1-Normal

<https://www.proteinatlas.org/ENSG00000276043-UHRF1/tissue/stomach#img>

UHRF1-Tumor

<https://www.proteinatlas.org/ENSG00000276043-UHRF1/pathology/stomach+cancer#img>
